# Global, regional, and national burdens of chronic kidney disease attributable to high body mass index from 1990 to 2021, with future forecasts up to 2050: a systematic analysis for the global burden of disease study 2021

**DOI:** 10.3389/fpubh.2025.1612300

**Published:** 2025-07-09

**Authors:** Jun Ying, Xiaolei Lan, Hanjing Zhou, Hongxia Li, Weixin Sheng, Jian Huang

**Affiliations:** Department of Nephrology, Affiliated Jinhua Hospital, Zhejiang University School of Medicine, Jinhua, China

**Keywords:** chronic kidney disease, global burden of disease, age-period-cohort, high body mass index, BAPC model

## Abstract

**Background:**

High body mass index (BMI) is a major modifiable risk factor for the development and progression of chronic kidney disease (CKD) through established mechanisms, including glomerular hyperfiltration and metabolic dysfunction. This study aimed to characterize the global, regional, and national temporal trends in CKD attributable to BMI from 1990 to 2021 and forecast trends up to 2050.

**Methods:**

Data were obtained from the Global Burden of Disease 2021 Study. Deaths and disability-adjusted life years (DALYs) for patients with CKD attributable to a high BMI were analyzed using age-standardized rates. The estimated annual percentage change was then calculated. Attribution was calculated as the product of the population-attributable fractions (PAFs) related to CKD deaths/DALYs and disease burden, with deaths/DALYs as the unit of measurement. Age-period cohort analysis was used to estimate the age, period, and cohort effects. Bayesian age-period-cohort modeling was used to predict the burden of CKD associated with high BMI from 2021 to 2050.

**Results:**

From 1990 to 2021, the burden of CKD attributable to BMI showed an increasing trend. Age-standardized death and DALY rates increased in all Sociodemographic Index regions from 1990 to 2021. At the national level, most countries have exhibited an increase in burden from 1990 to 2021. Among females, the 85–89 age group had the highest number of deaths (28,478), whereas among males, it was the 70–74 age group (25,270). Globally, high BMI is a risk factor for CKD, contributing to 27.3% of deaths. With respect to the age effect, deaths from CKD attributable to high BMI increased with age. The burden of CKD attributable to high BMI generally increases from 2021 to 2050.

**Conclusion:**

The burden of CKD attributable to high BMI increased significantly between 1990 and 2021. The growing global burden demands urgent and mechanistically informed health interventions that target the complex pathophysiology of obesity-related kidney diseases.

## 1 Introduction

Chronic Kidney Disease (CKD) is a clinical syndrome secondary to pathological changes in renal function and/or structure. It is characterized by irreversibility and progression and can advance to end-stage renal disease (1, 2). CKD is a major global public health issue, imposing substantial burdens on healthcare systems worldwide. In 2021, the CKD burden demonstrated an overall upward trend with regional disparities, with prevalence increasing in Africa and the Americas and declining in Asia, Europe, and Oceania (3). Risk factors for CKD include diabetes, hypertension, cardiovascular disease, metabolic syndrome, genetic predisposition, and drug-induced injury (4). CKD is also strongly correlated with adverse outcomes such as cardiovascular disease (5). Given this context, understanding the current status and burden of CKD is critical.

The global obesity rate is on a continuous upward trend, rising from 10.2% in 1990 to 20.8% in 2021, and the prevalence of obesity will increase to 34.0% by 2050 (6). Against the backdrop of rising global obesity rates, high body mass index (BMI) is linked to CKD incidence and progression. Latent trajectory analysis revealed that high BMI trajectories are correlated with subclinical renal injury in adults (7). In obesity, visceral adipose tissue secretes adipokines like increased leptin/free fatty acid and decreased adiponectin levels. Renin–angiotensin–aldosterone system activation and proximal tubule reabsorption weaken tubuloglomerular feedback, causing afferent arteriolar dilation, while Ang-II/aldosterone constrict efferents, leading to glomerular hyperfiltration and intraglomerular hypertension. This damages podocytes, causing obesity-related glomerulopathy and hypertension/lipotoxicity, which drives CKD progression (8). In 2021, high BMI contributed to 3.71 million deaths globally, with disease burdens disproportionately concentrated in low-and middle-income countries (9). Between 1990 and 2019, high BMI was the third risk factor for CKD-related death, resulting in a large increase in the CKD age-standardized death rate (ASDR) (10). Given its association with multiple comorbidities, addressing high BMI-related health impacts warrants urgent attention (11).

Epidemiological trends exhibit significant regional heterogeneity. Depth mining of high-quality, comprehensive health big data to analyze CKD burden and temporal patterns across regions can provide data support for medical resource allocation (12). This study utilizes the latest Global Burden of Disease Study (GBD) 2021 data and employs analytical methods such as age-period-cohort (APC) and Bayesian age-period-cohort (BAPC) models, along with the established GBD methodology incorporating spatiotemporal modeling, to ensure optimal comparability across different time periods and geographic regions. The objectives are to quantify the global, regional, and national burden of CKD attributable to high BMI in terms of deaths and DALYs from 1990 to 2021, identify demographic variations by geography, age, and gender, and develop future projections (2022–2050) to inform evidence-based CKD prevention strategies.

## 2 Methods

### 2.1 Data source

The GBD 2021 is a scaled-up, comprehensive, global disease epidemiology study designed to provide comprehensive data on the global burden of disease, death, age structure, DALYs, and the impact of health risk factors on health status. Completed by the University of Washington, USA, in collaboration with governments and non-governmental organizations (NGOs), the study uses the latest epidemiological methods and techniques to provide a comprehensive assessment of 369 diseases and injuries and 87 major risk factors in order to track trends over time, with data from 204 countries and territories covering the period 1990 to 2021. GBD employs a spatiotemporal Gaussian process regression methodology to leverage both temporal and spatial correlation patterns in epidemiological data. The present study extracted information from the database on the number of deaths, DALYs, ASDR, and age-standardized DALY rate for CKD attributable to high BMI and analyzed them according to time, age, sex, and Socio-demographic Index (SDI) for 204 countries and territories. The SDI is a composite indicator of the distribution of income per capita in a country or region, the average number of years of schooling of the population aged 15 and over, and the fertility rate of women aged under 25. On the basis of the SDI values, 204 countries and territories were categorized into five groups (high SDI regions, high-middle SDI regions, middle SDI regions, low-middle SDI regions, and low SDI regions). In order to analyze age-related trends, age was grouped at 5-year intervals to provide a clearer picture of trends in the burden of CKD attributable to high BMI in different age groups from 1990 to 2021. GBD data represent aggregated population-level estimates with established ethical oversight protocols.

### 2.2 Relevant definitions

High BMI was defined in this study as a BMI > 25 kg/m^2^ (13).

### 2.3 Statistical methods

Age-standardized rate (ASR): This measure is used to eliminate the effects of different age structures on statistical indicators and ensure comparability across countries and population sizes. The calculation of ASR was based on age-specific standardization using the World Standard Population from the GBD 2021 database. In this study, the ASR per 100,000 people was calculated using the following formula:


$$\mathrm{ASR}=\frac{\sum_{i=1}^{A}\text{aiwi}}{\sum_{i=1}^{A}\mathrm{wi}}\times 100000$$

*Ai* represents the age-specific rate for age group *i*; *wi* represents the corresponding population count for age group *i* in the standardized population; and *A* represents the total number of age groups. The results are presented as 95% uncertainty intervals (UI), with the 95% UI defined as the range between the 2.5th and 97.5th percentile values from 1,000 ordered samples.

Estimated Annual Percentage Change (EAPC): This is a value that quantifies the trend of rate changes within a specific interval. By fitting the natural logarithm of the ASR to years, the long-term trend in the ASR of the disease burden can be depicted. The formula used is Y = *α* + *β*X + e, where Y represents the natural logarithm of the ASR, X is the calendar year, α is the intercept, *β* represents the slope or trend, and e is the error term. EAPC is calculated as 100 × [exp(β) - 1], with the 95% confidence interval (CI) derived from a linear regression model. When both the EAPC and the lower limit of the 95% Confidence Interval (CI) are greater than 0, the trend is upward. When both the EAPC and the upper limit of the 95% CI are less than 0, the trend is downward. When 0 is included in the 95% CI, the trend is considered stable (14).

Attribution was calculated as the product of the population-attributable fractions (PAFs) related to CKD deaths/DALYs and disease burden, with deaths/DALYs as the unit.

The age-period-cohort (APC) model analysis reflects the contributions of factors such as age, period, and cohort effects to the burden in the development trend of CKD attributable to high BMI. The fitting of the APC model was performed using the Intrinsic Estimation (IE) method. The goodness-of-fit indices for the model were the Akaike Information Criterion (AIC) and Bayesian Information Criterion (BIC). This model reveals the age, period, and cohort effects through the division of different ages, periods, and cohorts. The calculation formula is as follows: The log-linear regression model is expressed as follows:


$$\log(Y_{i})=\mu +\alpha *{\mathrm{age}}_{i}+\beta *{\text{period}}_{i}+\gamma *{\text{cohort}}_{i}+\varepsilon$$

where *Yi* represents the prevalence, incidence, or DALY of CKD attributable to high BMI; *α*, *β*, and *γ* are coefficients for age, period, and cohort, respectively; *μ* is the intercept; and *ε* is the residual of the model (15). Age effects (longitudinal age curve) are age-specific death/DALY rates estimated by adjusting for period deviations and using a selected cohort as the reference. The transverse time curve refers to age-specific death/DALY rates estimated by adjusting for cohort bias and using a selected period as the reference. The cohort [or period rate ratio (RR)] represents the relative risk of the cohort (or period) relative to the reference cohort (or period), adjusted for age and nonlinear period (or cohort) effects (16). This study was statistically examined using the R-based APC model analysis toolkit developed by IHME, USA^1^.

The Bayesian age-period-cohort (BAPC) model considers changes in age, period, and cohort, assuming that these changes are similar over time (17). The BAPC model is an APC model within a Bayesian framework. It treats all unknown parameters as random parameters with appropriate prior distributions, no longer relying on explicit parameter settings, and can simultaneously consider additional parameters for non-structural differences to address the problem of large data dispersion. Simultaneously, it uses the prior information of unknown parameters and sample data to estimate the posterior distribution and infers unknown parameters based on the posterior distribution. This is different from classical statistics, which infers overall parameters using only sample data. The BAPC model can be expressed as ln(*λ_ij_*) = *μ* + *α_i_* + *β_j_* + *γ_k_* + *Z_ij_*, where *Z_ij_* represents non-structural variation parameters, and for non-structural variation parameters *Zij*, a Gaussian normal distribution with a mean of 0 is adopted. The BAPC model predicts the burden of CKD associated with high BMI from 2022 to 2050 based on sex. All statistical analyses and visualizations were performed using R software (version 4.4.3), and the APC and BAPC packages were used for the analysis.

## 3 Results

### 3.1 Global burden of CKD attributable to high BMI

**Figure 1:**
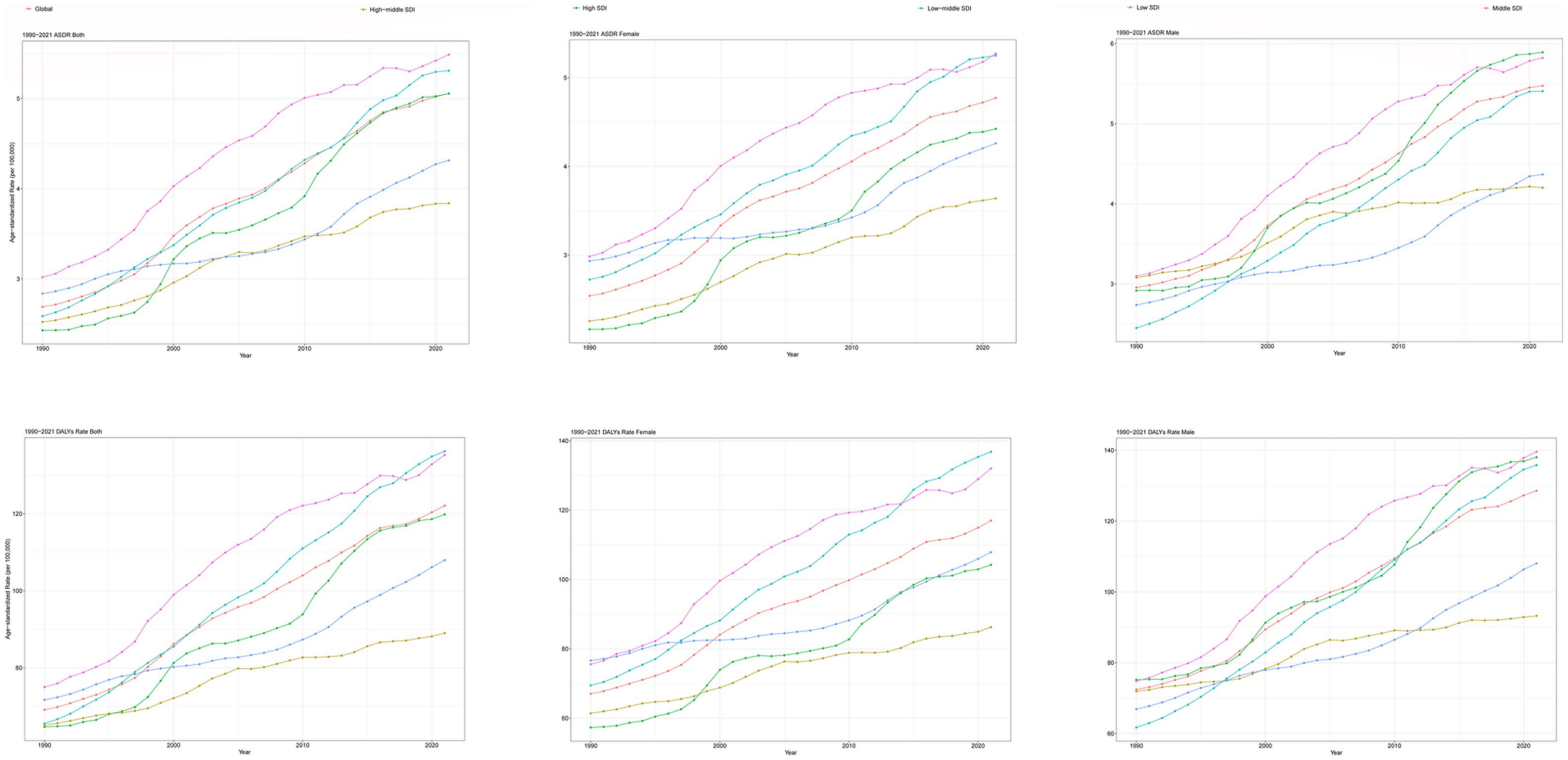
Trends in CKD attributable to high BMI, ASDR, and age-standardized DALYs rate from 1990 to 2021. CKD, chronic kidney disease; BMI, body mass index; ASDR, age-standardized death rate; DALYs, disability-adjusted life years.

The global deaths from CKD attributable to high BMI increased from 91,986 cases in 1990 (95% UI: 47,204–140,784) to 418,402 cases in 2021 (95% UI: 224,309–621,353). The ASDR rose from 2.69 per 100,000 people in 1990 (95% UI: 1.371–4.145) to 5.056 per 100,000 people in 2021 (95% UI: 2.696–7.514), with an EAPC of 2.25 (95% CI: 2.13–2.36) (Table 1; Figure 1). In addition, the number of global DALYs for CKD attributable to high BMI in 2021 totaled 10,422,561 (95% UI: 5,658,159–15,387,254), corresponding to an age-standardized DALY rate of 122.076 (95% UI: 66.249–180.184) per 100,000 people. The age-standardized DALY rate increased relative to 1990, with an EAPC of 1.98 (95% CI: 1.89–2.07) (Table 2; Figure 1).

**Table 1:** The global, regional burden of chronic kidney disease attributable to high body mass index: death and DALYs, 1990–2021.

Location	1990		2021		EAPC_CI
	Number	ASR	Number	ASR	
Deaths					
Global	91,986 (47204–140,784)	2.69 (1.371–4.145)	418,402 (224309–621,353)	5.056 (2.696–7.514)	2.25 (2.13–2.36)
SDI					
High-middle SDI	21,040 (10946–32,112)	2.523 (1.312–3.873)	73,547 (39495–110,229)	3.839 (2.057–5.757)	1.48 (1.4–1.56)
High SDI	26,663 (13925–39,253)	2.431 (1.273–3.578)	121,231 (63489–179,474)	5.055 (2.755–7.385)	2.75 (2.6–2.9)
Low-middle SDI	13,326 (6887–21,134)	2.587 (1.313–4.148)	69,370 (36960–104,792)	5.309 (2.781–8.041)	2.46 (2.39–2.53)
Low SDI	5,523 (2778–9,323)	2.836 (1.387–4.801)	18,981 (9372–30,895)	4.314 (2.094–6.982)	1.3 (1.18–1.42)
Middle SDI	25,293 (12768–40,494)	3.019 (1.484–4.923)	134,842 (72232–204,349)	5.487 (2.916–8.347)	2.08 (1.9–2.27)
World Bank region					
High income	2,434 (1176–3,600)	1.602 (0.773–2.37)	8,485 (4096–12,838)	2.697 (1.327–4.043)	2.32 (2.13–2.5)
Middle income	9,166 (4602–15,172)	1.742 (0.848–2.946)	49,019 (25362–77,667)	3.446 (1.757–5.529)	2.11 (2–2.21)
Low income	1,334 (611–2,298)	1.695 (0.775–2.927)	6,289 (3090–10,662)	3.012 (1.46–5.082)	1.85 (1.74–1.96)
Continents					
Africa	12,341 (6488–20,028)	5.338 (2.761–8.742)	55,809 (29663–83,845)	10.528 (5.475–16.011)	2.24 (2.21–2.27)
America	26,394 (14148–37,672)	4.455 (2.395–6.357)	142,893 (79322–203,319)	10.416 (5.815–14.791)	3.04 (2.75–3.32)
Asia	31,406 (15295–53,475)	1.911 (0.902–3.279)	153,550 (78428–243,738)	3.274 (1.662–5.223)	1.76 (1.72–1.8)
Europe	21,286 (11172–32,032)	2.147 (1.128–3.235)	64,463 (32916–96,672)	3.478 (1.794–5.176)	1.94 (1.84–2.04)
WHO regions					
African Region	7,882 (3887–12,936)	4.282 (2.073–7.075)	33,653 (17413–52,462)	8.174 (4.068–12.973)	2 (1.93–2.06)
Eastern Mediterranean Region	10,017 (5459–15,934)	6.544 (3.465–10.511)	48,859 (27407–71,461)	12.832 (7.047–18.818)	2.26 (2.24–2.29)
European Region	21,884 (11502–32,935)	2.137 (1.124–3.217)	67,087 (34221–100,385)	3.533 (1.82–5.264)	1.99 (1.89–2.1)
Region of the Americas	26,394 (14148–37,672)	4.455 (2.395–6.357)	142,893 (79322–203,319)	10.416 (5.815–14.791)	3.04 (2.75–3.32)
South-East Asia Region	7,239 (3505–12,294)	1.14 (0.545–1.928)	42,774 (20659–70,890)	2.545 (1.21–4.242)	2.61 (2.55–2.67)
Western Pacific Region	17,399 (8312–31,279)	1.942 (0.918–3.481)	78,909 (39721–129,904)	2.889 (1.441–4.734)	1.31 (1.23–1.38)
GBD super regions					
Andean Latin America	1,540 (791–2,270)	8.196 (4.141–12.201)	8,421 (4742–12,292)	14.717 (8.232–21.553)	1.88 (1.53–2.23)
Australasia	513 (259–799)	2.335 (1.179–3.622)	2,295 (1234–3,395)	3.64 (1.979–5.354)	2.07 (1.81–2.34)
Caribbean	1,190 (641–1804)	4.911 (2.634–7.541)	5,079 (2811–7,586)	9.328 (5.171–13.915)	2.68 (2.49–2.86)
Central Asia	463 (232–744)	0.995 (0.504–1.603)	2,429 (1230–3,844)	3.138 (1.582–5.019)	3.31 (2.82–3.81)
Central Europe	4,274 (2337–6,398)	3.06 (1.682–4.582)	7,746 (4296–11,527)	3.299 (1.822–4.91)	0.4 (0.21–0.59)
Central Latin America	5,431 (2817–8,270)	7.343 (3.777–11.229)	37,003 (21163–53,500)	15.006 (8.524–21.751)	2.78 (2.25–3.31)
Central Sub-Saharan Africa	980 (493–1,647)	5.364 (2.651–9.064)	4,082 (1990–7,104)	9.19 (4.505–16.021)	1.57 (1.44–1.7)
East Asia	12,455 (5678–23,610)	1.885 (0.859–3.561)	57,532 (28921–96,865)	2.914 (1.444–4.879)	1.38 (1.28–1.48)
Eastern Europe	1758 (906–2,736)	0.646 (0.333–1)	5,571 (2968–8,288)	1.566 (0.836–2.329)	2.65 (2.28–3.03)
Eastern Sub-Saharan Africa	1973 (927–3,497)	3.091 (1.392–5.416)	7,402 (3541–12,374)	5.225 (2.437–8.756)	1.56 (1.49–1.64)
High-income Asia Pacific	3,635 (1751–5,865)	2.068 (0.98–3.393)	12,978 (6099–21,292)	1.966 (0.935–3.193)	−0.2 (−0.31–−0.09)
High-income North America	10,811 (5808–15,053)	2.974 (1.609–4.129)	65,218 (35049–93,596)	9.259 (5.07–13.118)	4.09 (3.87–4.3)
North Africa and the Middle East	11,914 (6417–18,476)	8.653 (4.595–13.711)	56,206 (31393–81,076)	14.651 (7.978–21.36)	1.86 (1.71–2.01)
Oceania	87 (41–151)	3.551 (1.595–6.383)	349 (154–589)	5.579 (2.514–9.511)	1.35 (1.21–1.49)
South Asia	5,580 (2828–9,532)	1.074 (0.529–1.851)	32,710 (16889–53,590)	2.36 (1.196–3.93)	2.55 (2.47–2.62)
Southeast Asia	4,327 (1974–7,333)	1.846 (0.862–3.159)	25,018 (11359–41,260)	4.114 (1.822–6.865)	2.66 (2.57–2.75)
Southern Latin America	3,379 (1788–4,903)	7.84 (4.143–11.418)	8,273 (4557–12,191)	9.08 (5.034–13.354)	0.73 (0.37–1.1)
Southern Sub-Saharan Africa	993 (490–1,637)	4.078 (1.993–6.794)	5,469 (2810–8,438)	11.121 (5.601–17.168)	3.25 (2.8–3.7)
Tropical Latin America	4,497 (2476–6,494)	5.514 (2.98–8.074)	20,289 (11566–27,902)	8.124 (4.606–11.221)	1.28 (1.09–1.47)
Western Europe	12,769 (6405–19,413)	2.134 (1.073–3.255)	41,284 (19854–64,192)	3.228 (1.561–4.979)	1.85 (1.68–2.03)
Western Sub-Saharan Africa	3,417 (1646–5,538)	4.672 (2.244–7.609)	13,045 (6411–20,612)	8.172 (3.936–12.901)	1.66 (1.58–1.73)

DALYs, disability-adjusted life years; SDI, social demographic index.

**Table 2:** The global, regional burden of chronic kidney disease attributable to high body mass index: DALYs, 1990–2021.

Location	1990		2021		EAPC_CI
	Number	ASR	Number	ASR	
DALYs					
Global	2,670,085 (1367921–4,086,227)	69.129 (35.065–106.004)	10,422,561 (5658159–15,387,254)	122.076 (66.249–180.184)	1.98 (1.89–2.07)
SDI					
High-middle SDI	614,301 (316143–936,793)	65.112 (33.262–99.786)	1,724,597 (925553–2,563,740)	89.036 (47.481–132.5)	1.09 (1.02–1.16)
High SDI	704,468 (368717–1,028,330)	64.685 (33.966–94.568)	2,461,009 (1365309–3,490,312)	119.828 (68.686–167.366)	2.26 (2.15–2.38)
Low-middle SDI	403,769 (208015–634,935)	65.552 (33.753–104.303)	2,013,408 (1064386–3,012,735)	136.218 (71.428–204.328)	2.5 (2.43–2.57)
Low SDI	167,824 (83818–281,187)	71.683 (35.635–119.854)	586,369 (292058–940,091)	107.945 (52.876–174.769)	1.24 (1.15–1.33)
Middle SDI	775,653 (391726–1,247,666)	74.991 (37.296–120.405)	3,626,442 (1961571–5,392,067)	135.252 (72.539–202.011)	2.03 (1.84–2.21)
World Bank region					
High income	70,762 (36175–103,986)	47.651 (24.144–70.053)	181,422 (93257–264,985)	66.529 (35.202–96.255)	1.44 (1.32–1.56)
Middle income	310,593 (152753–509,353)	47.847 (23.404–78.188)	1,592,025 (802695–2,473,619)	97.33 (48.692–151.72)	2.28 (2.19–2.37)
Low income	39,991 (18405–68,338)	43.688 (20.212–75.138)	190,138 (94156–314,698)	77.949 (38.38–130.273)	1.94 (1.88–2.01)
Continents					
Africa	347,938 (182719–552,019)	123.51 (64.82–196.612)	1,525,656 (826173–2,269,346)	231.837 (122.001–350.756)	2.06 (2.02–2.1)
America	719,293 (389317–1,024,067)	118.208 (63.975–167.791)	3,296,205 (1901713–4,614,985)	248.528 (144.223–346.8)	2.62 (2.37–2.88)
Asia	967,384 (469485–1,630,518)	48.368 (23.227–81.597)	4,249,528 (2172463–6,711,586)	84.287 (43.075–133.587)	1.86 (1.81–1.91)
Europe	621,129 (329556–917,339)	61.725 (32.864–91.199)	1,315,863 (703340–1,909,101)	80.62 (43.813–115.837)	1.01 (0.97–1.06)
WHO regions					
African Region	232,198 (116098–381,336)	103.225 (51.188–169.728)	963,965 (506811–1,478,325)	184.225 (95.677–285.083)	1.78 (1.71–1.84)
Eastern Mediterranean Region	275,713 (154284–416,536)	152.006 (84.578–234.859)	1,334,162 (756833–1,949,202)	287.394 (161.38–422.689)	2.13 (2.09–2.16)
European Region	649,734 (344630–958,730)	62.372 (33.226–92.057)	1,412,550 (755529–2,051,971)	84.113 (45.657–121.066)	1.1 (1.06–1.15)
Region of the Americas	719,293 (389317–1,024,067)	118.208 (63.975–167.791)	3,296,205 (1901713–4,614,985)	248.528 (144.223–346.8)	2.62 (2.37–2.88)
South-East Asia Region	261,768 (124617–442,197)	34.368 (16.215–58.172)	1,400,255 (671180–2,286,728)	74.456 (35.625–121.684)	2.6 (2.54–2.65)
Western Pacific Region	501,872 (237307–907,657)	45.874 (21.708–82.32)	1,928,324 (974985–3,121,377)	68.875 (34.531–111.453)	1.38 (1.3–1.47)
GBD super regions					
Andean Latin America	38,537 (20582–55,186)	186.821 (99.347–269.384)	189,671 (111879–269,060)	320.22 (188.474–454.36)	1.71 (1.39–2.03)
Australasia	12,321 (6423–18,563)	53.973 (28.181–81.359)	42,604 (23911–60,100)	76.157 (43.493–106.957)	1.48 (1.29–1.66)
Caribbean	33,155 (18161–47,971)	126.292 (69.093–183.857)	125,996 (72499–182,325)	234.81 (135.396–339.311)	2.55 (2.39–2.71)
Central Asia	33,131 (17919–49,692)	68.68 (37.394–102.789)	109,844 (57968–166,719)	127.697 (67.686–193.213)	1.84 (1.57–2.11)
Central Europe	129,365 (71069–190,551)	88.996 (48.978–131.399)	193,117 (107518–281,847)	89.32 (50.245–130.684)	0.17 (0.05–0.29)
Central Latin America	160,409 (84342–240,900)	186.213 (97.626–279.016)	1,000,701 (584007–1,445,868)	391.241 (227.136–565.476)	2.78 (2.28–3.29)
Central Sub-Saharan Africa	30,772 (15408–50,944)	132.203 (66.316–219.034)	127,980 (63569–217,363)	218.665 (108.957–371.982)	1.45 (1.34–1.56)
East Asia	371,394 (166747–709,823)	44.839 (20.233–84.999)	1,459,584 (735849–2,378,609)	68.237 (34.246–112.209)	1.42 (1.3–1.54)
Eastern Europe	104,202 (55033–156,159)	38.908 (20.311–58.481)	195,886 (111943–283,093)	57.202 (32.547–82.885)	0.95 (0.82–1.09)
Eastern Sub-Saharan Africa	55,257 (25062–95,096)	72.368 (33.485–127.01)	206,435 (99743–337,029)	117.806 (56.435–195.005)	1.43 (1.36to1.49)
High-income Asia Pacific	90,151 (44364–146,537)	46.596 (22.797–76.065)	224,775 (106377–364,719)	43.854 (21.26–70.036)	−0.16 (−0.25–-0.07)
High-income North America	282,076 (154431–391,817)	81.262 (44.787–112.202)	1,337,866 (769238–1,845,875)	209.796 (123.683–284.456)	3.4 (3.2–3.6)
North Africa and the Middle East	315,238 (175791–483,479)	190.678 (104.685–295.367)	1,408,667 (811435–2,033,498)	311.831 (178.633–449.15)	1.72 (1.63–1.8)
Oceania	2,989 (1395–5,061)	95.793 (43.852–164.394)	10,998 (4923–17,896)	140.729 (62.796–233.047)	1.17 (1.03–1.3)
South Asia	205,937 (100659–348,113)	33.153 (16.141–55.661)	1,135,827 (572346–1,826,420)	72.966 (36.465–118.139)	2.65 (2.59–2.71)
Southeast Asia	137,993 (60978–236,176)	50.449 (22.695–86.09)	732,323 (335705–1,219,007)	106.922 (48.703–176.805)	2.54 (2.44–2.64)
Southern Latin America	75,156 (41352–106,864)	165.385 (90.153–235.43)	160,813 (90310–230,298)	183.136 (103.232–260.251)	0.58 (0.26–0.9)
Southern Sub-Saharan Africa	33,500 (17239–52,623)	115.806 (59.705–183.722)	158,454 (84635–239,419)	268.331 (140.76–408.082)	2.73 (2.35–3.11)
Tropical Latin America	141,145 (78383–201,580)	148.607 (81.448–214.842)	509,000 (293912–689,733)	197.792 (113.713–269.46)	0.83 (0.64–1.02)
Western Europe	318,605 (161100–479,304)	55.174 (27.821–82.989)	702,642 (345130–1,050,965)	66.641 (33.4–99.212)	0.84 (0.75–0.93)
Western Sub-Saharan Africa	98,752 (49143–159,821)	111.986 (54.704–181.562)	389,379 (199598–615,269)	188.716 (94.715–297.393)	1.56 (1.48–1.63)

DALYs, disability-adjusted life years; SDI, social demographic index.

### 3.2 Regional burden of CKD attributable to high BMI

At the SDI level, the ASDR and age-standardized DALY rates for CKD attributable to high BMI increased in all SDI regions from 1990 to 2021 (Tables 1, 2; Figure 1). In the high-middle SDI regions, the lowest ASDR [3.839 (95% UI: 2.057–5.757) per 100,000 people], age-standardized DLAY rate [89.036 (95% UI: 47.481–132.5) per 100,000 people] for CKD attributable to high BMI in 2021, and lowest increase of age-standardized DALYs rates for CKD attributable to high BMI [EAPC: 1.09 (95% CI: 1.02–1.16)] from 1990 to 2021 (Tables 1, 2; Figure 1). The highest ASDR, age-standardized DLAY rate for CKD attributable to high BMI in 2021, was in the low-middle SDI regions, and the highest increase in ASDR for CKD attributable to high BMI was in middle SDI regions (Tables 1, 2; Figure 1). In terms of ASDR, from 1990 to 2021, the fastest rise was in the high-SDI regions, with an EAPC of 2.75 (95% CI: 2.6–2.9); the slowest rise was in the low-SDI regions, with an EAPC of 1.3 (95% CI: 1.18–1.42) (Table 1; Figure 1).

At the continental level, the burden of CKD attributable to high BMI on all four continents increased from 1990 to 2021 (Tables 1, 2). Asia showed the lowest ASDR [3.274 (95% UI: 1.662–5.223) per 100,000 people] in 2021 and the lowest increase in ASDR [EAPC: 1.76 (95% CI: 1.72–1.8)] for CKD attributable to high BMI from 1990 to 2021, while in the case of age-standardized DALYs rate, the lowest occurred in Europe (Tables 1, 2). America had the highest increase in ASDR [EAPC: 3.04 (95% CI: 2.75–3.32)] and age-standardized DLAY rate [EAPC: 2.62 (95% CI: 2.37–2.88)] of CKD attributable to high BMI from 1990 to 2021 and the highest age-standardized DLAY rate [248.528 (95% UI: 144.223–346.8) per 100,000 people] in 2021 (Tables 1, 2). Africa reached the highest ASDR [10.528 (95% UI: 5.475–16.011) per 100,000 people] of CKD attributable to high BMI among the four states in 2021 (Table 1).

At the World Health Organization (WHO) regional level, the burden in all regions showed an upward trend from 1990 to 2021 (Tables 1, 2). The Eastern Mediterranean region had the highest burden of CKD attributable to high BMI in 2021 (Tables 1, 2). The regions of the Americas showed the highest increase in ASDR and age-standardized DLAY rates of CKD, attributable to high BMI from 1990 to 2021. At the World Bank region level, the burden in all regions showed an upward trend from 1990 to 2021. The lowest ASDR and age-standardized DLAY rates of CKD attributable to high BMI in 2021 were found in high-income regions (Tables 1, 2). In the GBD super-regions, except for the high-income Asia–Pacific, the burden in all other regions showed an upward trend. High-income North America had the highest increase in ASDR and age-standardized DLAY rate of CKD attributable to high BMI from 1990 to 2021, and the high-income Asia Pacific had the lowest increase in ASDR and age-standardized DLAY rate of CKD attributable to high BMI from 1990 to 2021 (Tables 1, 2).

### 3.3 National burden of CKD attributable to high BMI

**Figure 2:**
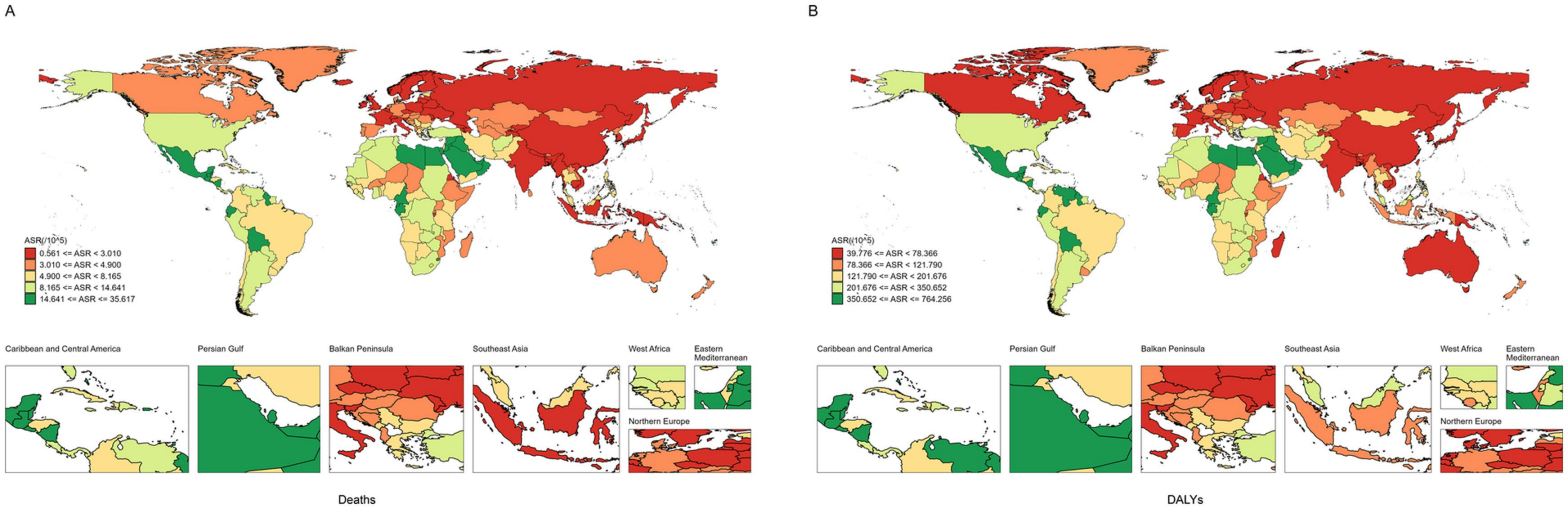
The ASR of CKD attributable to high BMI in 204 countries and territories. **(A)** Deaths. **(B)** DALYs. ASR, age-standardized rates; CKD, chronic kidney disease; BMI, body mass index; DALYs, disability-adjusted life years.

**Figure 3:**
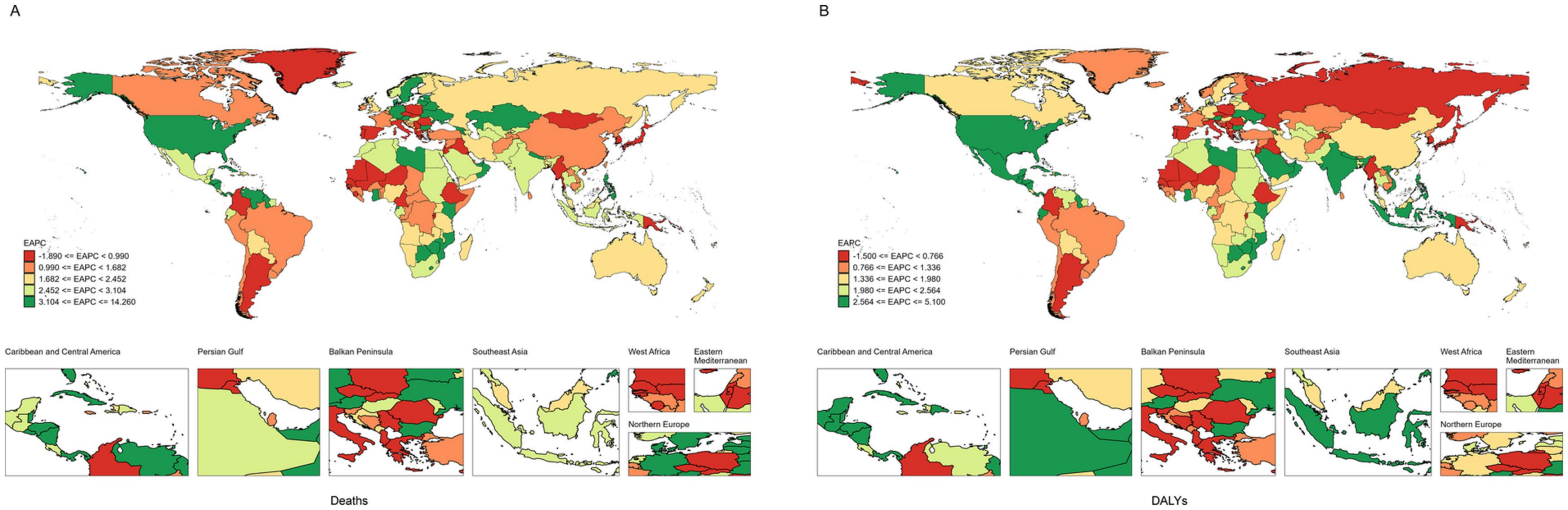
The EAPC of CKD attributable to high BMI in 204 countries and territories. **(A)** Deaths. **(B)** DALYs. EAPC, Estimated annual percentage change; CKD, chronic kidney disease; BMI, body mass index; DALYs, disability-adjusted life years.

At the national level, most countries showed an upward trend in burden from 1990 to 2021 (Supplementary Table S1; Figures 2, 3). Ukraine had the lowest ASDR [95% UI: 0.561 (0.275–0.958) per 100,000 people] of CKD attributable to high BMI in 2021 and experienced the highest ASDR growth of CKD attributable to high BMI from 1990 to 2021, with an EAPC of 14.26 (95% CI: 12.24 to 16.31) (Supplementary Table S1; Figures 2, 3). Poland had the lowest increase in ASDR and age-standardized DLAY rate of CKD attributable to high BMI from 1990 to 2021, with EAPCs of −1.89 (95% CI: −2.5–−1.27) and −1.5 (95% CI: −1.89–−1.11), respectively. In addition, Saudi Arabia showed the highest ASDR of CKD attributable to high BMI in 2021 [35.617 (95% UI: 19.801–53.057) per 100,000 people], and the same result was found in American Samoa in age-standardized DLAY rate [668.9 (95% UI: 321.389–1029.317) per 100,000 people]. The highest age-standardized DLAY rate of CKD attributable to high BMI from 1990 to 2021 was observed in Lesotho, with an EAPC of 5.1 (95% CI: 4.58–5.61). Finland had the lowest age-standardized DLAY rate of CKD attributable to high BMI in 2021, with an age-standardized DLAY rate of 39.776 (95% UI: 19.594–61.124) per 100,000 people (Supplementary Table S1; Figure 2).

### 3.4 CKD burden attributable to high BMI based on sex and age

**Figure 4:**
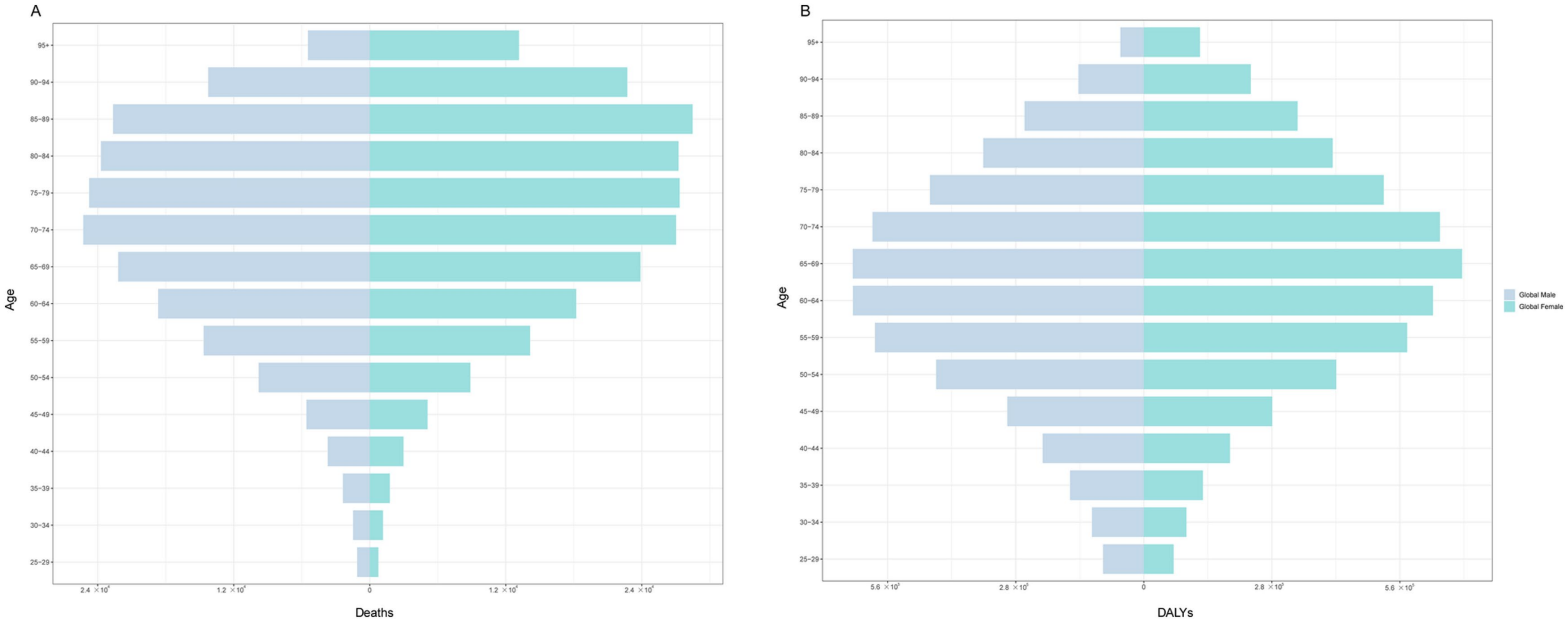
The burden of CKD attributable to high BMI by age and sex in 2021. **(A)** Prevalence. **(B)** Deaths. **(C)** DALYs. CKD, chronic kidney disease; BMI: body mass index; DALYs, disability-adjusted life years.

**Figure 5:**
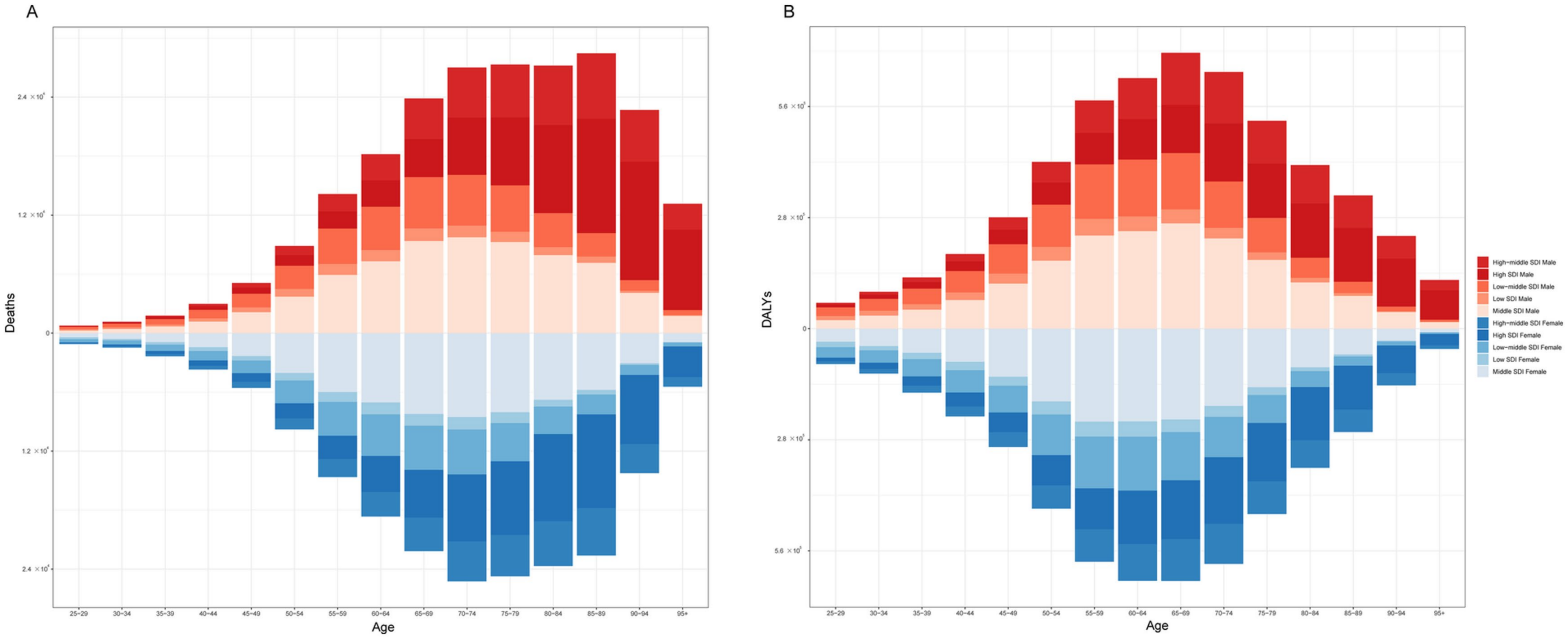
The burden of CKD attributable to high BMI by age and sex across SDI regions in 2021. **(A)** Deaths. **(B)** DALYs. CKD, chronic kidney disease; BMI, body mass index; SDI, socio-demographic index; DALYs, disability-adjusted life years.

Figure 4 shows that among females, the age group of 85–89 years had the highest number of deaths, at 28,478. Among males, the age group of 70–74 years had the highest number of deaths (25,270). In terms of the number of DALYs, the highest number was in the 65–69 age group, regardless of sex, with 695,876 females and 636,394 males. When stratified by SDI, males and females had the highest number of deaths and DALYs in the middle-SDI region (Figure 5).

### 3.5 Attribution of high BMI

**Figure 6:**
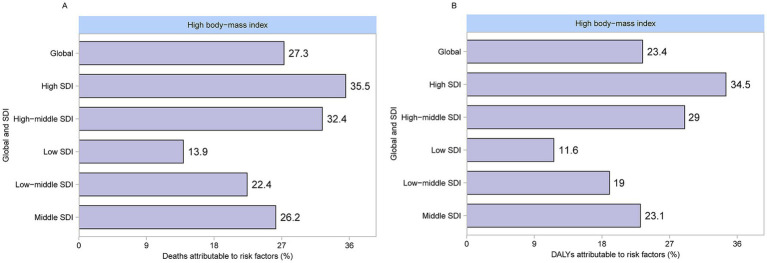
Proportion of CKD attributable to high BMI deaths and DALYs in 2021. **(A)** Deaths. **(B)** DALYs. CKD, chronic kidney disease; BMI, body mass index; DALYs, disability-adjusted life years.

Figure 6 shows the attribution of high BMI to CKD. Globally, among the risk factors for CKD, a high BMI contributes to 23.4% of deaths. The proportion of CKD deaths attributable to high BMI varied significantly across different SDI levels. The low-SDI regions had the lowest share at 13.9%, followed by low-middle SDI regions at 22.4%, middle SDI regions at 26.2%, high-middle SDI regions at 32.4%, and high SDI regions at 35.5%. Globally, among the risk factors for CKD, high BMI contributed to 27.3% of the DALYs. The proportion of CKD-related DALYs attributable to high BMI varied across different SDI regions. High-SDI regions recorded the highest share at 34.5%, whereas low-SDI regions had the lowest at 11.6%.

### 3.6 APC modeling of CKD attributable to high BMI

**Figure 7:**
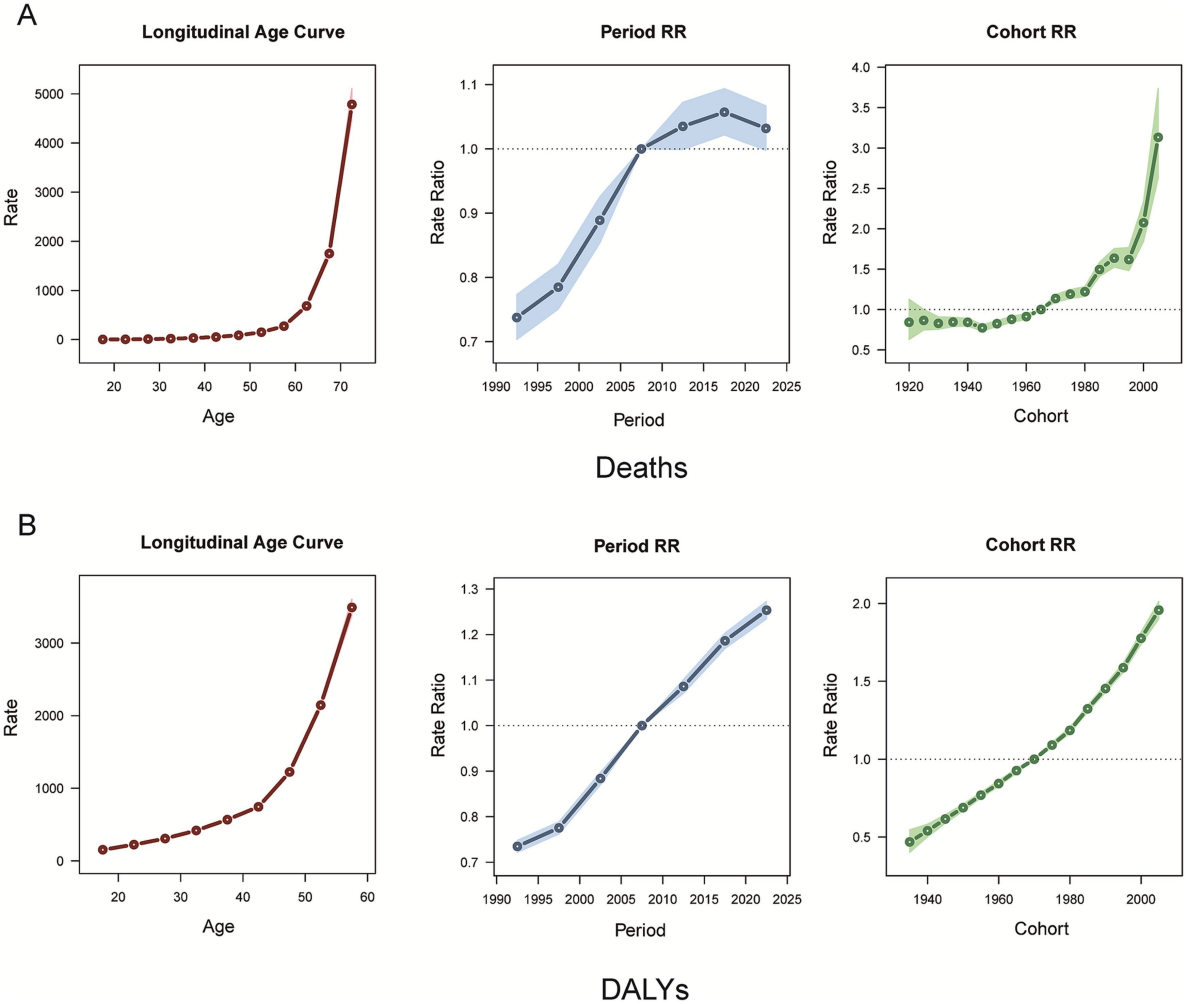
Age, period, and cohort effects on CKD attributable to high BMI deaths and DALYs during 1990–2021. **(A)** Deaths. **(B)** DALYs. CKD, chronic kidney disease; BMI, body mass index; DALYs, disability-adjusted life years.

For the age effect, death from CKD attributable to high BMI increased with age, with a slow increase before the age of 60 years, followed by a rapid acceleration thereafter (Figure 7A), while the DALY rate also showed an overall increasing age-related trend (Figure 7B). Regarding the period effect, the period RR of deaths from CKD attributable to high BMI increased for cohorts born before 2015–2020, whereas for cohorts born after 2015–2020, it gradually decreased with increasing years of birth (Figure 7A). The RR period of the DALYs rate of CKD attributable to high BMI has been observed to increase consistently (Figure 7B). From 1990 to 2021, the cohort RR of deaths and DALY rate of CKD attributable to high BMI showed a consistent upward trend (Figures 7A,B).

### 3.7 Future forecasts of the burden of CKD associated with high BMI

**Figure 8:**
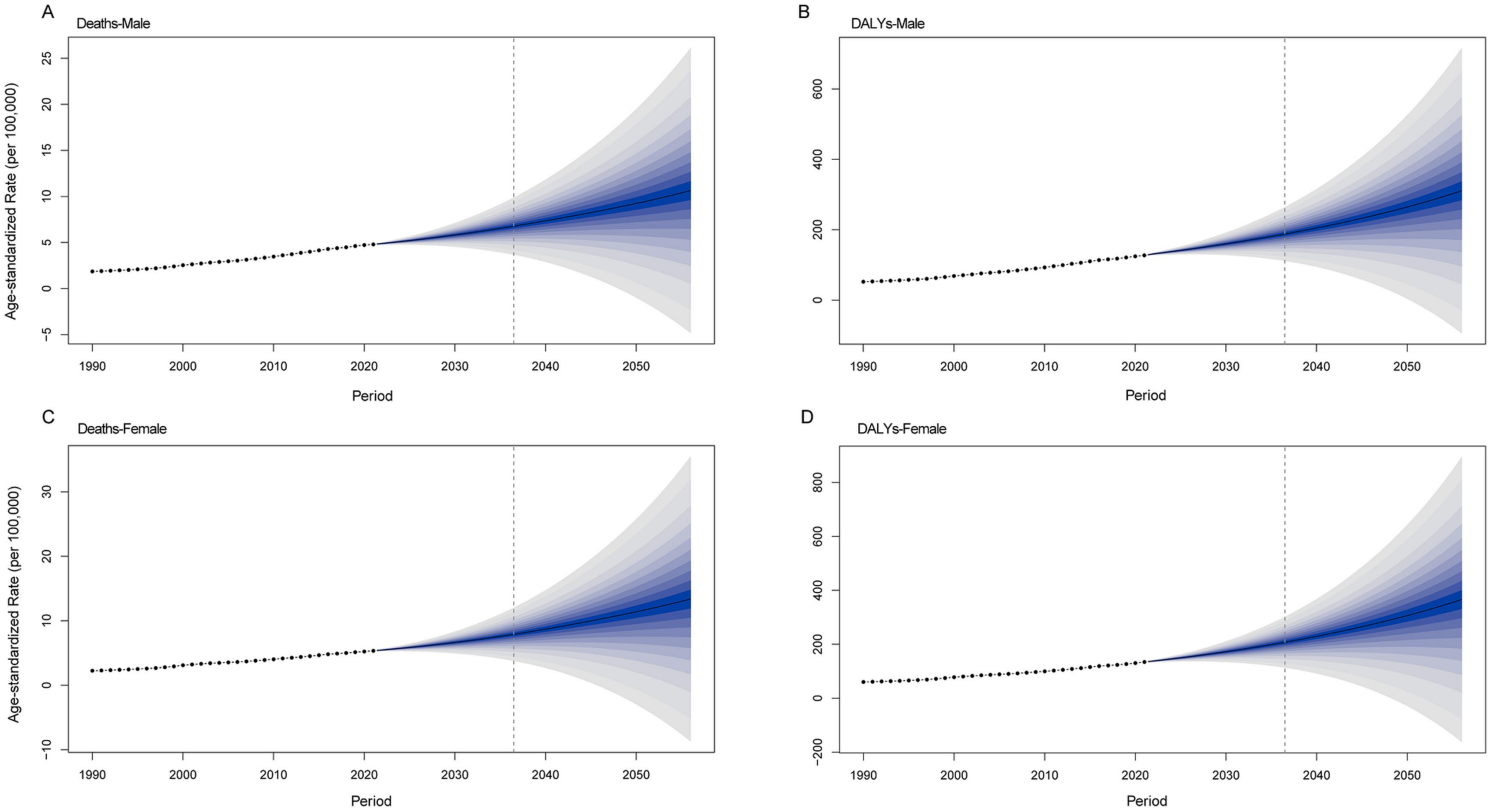
Future projections of the global burden of CKD attributable to high BMI by sex. **(A)** Male deaths. **(B)** Female deaths. **(C)** Male DALYs. **(D)** Female DALYs. CKD, chronic kidney disease; BMI, body mass index; DALYs, disability-adjusted life years.

Figure 8 shows an increasing trend in the burden of CKD attributable to high BMI in both males and females. The ASDR is projected to increase from 4.8 per 100,000 people in 2021 to 9.24 per 100,000 people in 2050 in the male population. In the female population, the ASDR will be 11.4 per 100,000 people in 2050. The age-standardized DALY rate for males is projected to increase from 127.14 per 100,000 people in 2021 to 264.45 per 100,000 people in 2050. For females, the age-standardized DALY rate will increase to 306.52 per 100,000 people.

## 4 Discussion

This study combined 204 countries and territories to assess global, regional, and national patterns and trends in the burden of CKD attributable to high BMI over a 30-year period and found that the burden of CKD attributable to high BMI was rising at the global level. In 2021, 619,494 people died worldwide because of high BMI, more than double the number in 1990. Additionally, 10,422,561 DALYs were lost due to CKD attributable to high BMI, approximately twice as many as in 1990. This result compares with a worldwide population ratio of 1.49 in 2021 compared with 1990, highlighting the progressive increase in the burden of CKD attributable to high BMI.

Data from the WHO for 2022 showed that approximately 890 million adults worldwide were obese (18). In Europe, 59% of adults and nearly one-third of children are overweight or obese (19). At present, approximately half of Chinese adults and one-fifth of Chinese children are overweight or obese, and by 2030, the prevalence of overweight or obesity among Chinese adults is expected to reach 65.3% (20). Being overweight and obesity are risk factors for CKD (21). Obesity leads to CKD through multiple mechanisms: hemodynamic changes cause abnormal pressure within the kidneys, metabolic disorders trigger insulin and lipid metabolism disorders, and imbalances in adipocytokines, along with oxidative stress and inflammatory responses, exacerbate kidney damage (22, 23). Meanwhile, mechanical pressure resulting from the accumulation of abdominal and renal fat also promotes the development of CKD (24). A study found that compared with the non-obese group (BMI < 25), the obese group (BMI ≥ 25) had a higher risk of CKD (OR: 1.323, 95% CI: 1.250–1.400), and this risk showed an upward trend after adjustment for confounding factors (25). These findings align with established evidence that obesity is one of the strongest modifiable risk factors for incident CKD (26). In addition, the prevalence of obesity in patients with CKD is relatively high. Relevant studies have found that 65% of patients with CKD were obese (27). These findings may explain the increased burden of diabetes in patients with CKD attributable to high BMI.

In this study, we found that Poland had the lowest increase in the ASDR and age-standardized DALY rate of CKD, which was attributable to high BMI from 1990 to 2021. Poland has a relatively well-developed healthcare system with a high rate of universal healthcare coverage, which allows for early detection and intervention of CKD (28). Limited access to renal replacement therapy may be a reason for the deterioration in mortality rates caused by kidney diseases (29). The availability of renal replacement therapy in Poland is similar to that in other EU countries (30). Polish patients were satisfied with the information they received regarding transplantation and in-hospital hemodialysis (31). Moreover, Polish patients were more frequently rescheduled to receive adequate consultations or rehabilitation than average European patients (31). However, Saudi Arabia had the highest ASDR for CKD, attributable to high BMI in 2021. Saudi Arabia’s diet has shifted to a Western diet and is low in vitamins, whereas the Western diet is high in fat, salt, and sugar, leading to high rates of obesity (32, 33). Differences in the burden of CKD attributable to high BMI between countries reflect broader patterns of health inequity in CKD access and outcomes documented globally (34) and population-specific factors, including genetic susceptibility, environmental exposures, and healthcare access patterns (35). In the near future, individuals and policymakers will need to work together to mitigate the effects of high BMI on CKD, both in terms of healthcare policies and diets.

In the GBD super regions, the high-income Asia Pacific had the lowest increase in ASDR and age-standardized DLAY rate of CKD attributable to high BMI from 1990 to 2021. High-income Asia-Pacific countries included Japan, Singapore, and South Korea in the GBD study. In Singapore, the prevalence rates of overweight and obesity are relatively low (36). The Health Promotion Board of Singapore organized a national weight management program that encompasses nutrition education, physical activities, mental health counseling courses, and periodic assessments (37). This program is widely popular and effective for short-term weight loss. Additionally, evidence-based recommendations on multidisciplinary management (including diet, physical exercise, pharmacotherapy, and surgery) of overweight and obesity have been published (38).

In terms of age, the burden of CKD attributable to high BMI was higher in the older adult population. In the older adult, due to the natural decline of renal function, changes in fat distribution, and the frequent coexistence of diseases such as hypertension and diabetes, coupled with reduced physical activity and weakened drug metabolism ability, a high BMI is more likely to increase the burden on the kidneys (39, 40). In addition, aging is accompanied by a decrease in muscle mass and an increase in the proportion of adipose tissue, leading to an underestimation of obesity by BMI (41). Visceral fat accumulation directly damages the glomeruli by secreting leptin, causing adiponectin imbalance, and releasing pro-inflammatory factors (such as IL-6 and TNF-*α*). Meanwhile, reduced muscle mass decreases creatinine production, thereby masking the early decline in renal function (42). Therefore, the situation of CKD attributable to BMI in the older adult population deserves more attention, and it is necessary to strengthen nursing care plans.

This study also projected the burden of CKD attributable to high BMI in 2050, revealing an increasing trend among both males and females. Hu et al. predicted the burden of CKD caused by type 2 diabetes mellitus and found a rising trend by 2036 (43). Another study projected that by 2040, the global CKD burden attributable to dietary risks would also show an upward trajectory. Future projections of CKD burden consistently indicate an increasing trend. These forecasts align with the contemporaneous predictions regarding obesity, which also reflect an increasing burden. These studies collectively highlight the substantial burden of CKD and underscore the urgent need for effective interventions to mitigate this burden.

Despite the coverage of 204 countries and territories, this study has several limitations that must be addressed. First, this study was based on GBD 2021, which is not derived from raw data but uses a variety of mathematical models and combines a large amount of data to make predictions about the burden of disease; therefore, the results may be biased. Second, although GBD 2021 covers 204 countries and regions, variations in data availability and quality across regions may have affected the accuracy of the analysis. Particularly in areas with low SDI, insufficient medical resources and limitations in data collection may lead to underestimation of CKD cases. Finally, when predicting future burdens, this study failed to consider the impact of other factors.

## 5 Conclusion

This study highlights the significant and growing burden of CKD associated with high BMI globally. At the regional level, the burden of CKD attributable to high BMI trended upward in all SDI regions. At the national level, Poland had the lowest increase in the ASDR and age-standardized DALY rate of CKD, attributable to high BMI from 1990 to 2021. The burden of CKD attributable to high BMI was higher in the older adult population. In light of these results, it is crucial to continuously increase investment in research and prevention initiatives for CKD associated with high BMI, such as drawing on multidisciplinary planning experience, continuously increasing investment in related research and prevention, and adopting an integrated health framework that combines obesity prevention with non-communicable disease control and healthy aging.

## Data Availability

Publicly available datasets were analyzed in this study. This data can be found here: https://www.healthdata.org/research-analysis/gbd.

## Footnotes

^1^ http://analysistools.cancer.gov/apc/
