# Supplementary material for: Global, regional, and national burdens of chronic kidney disease attributable to high body mass index from 1990 to 2021, with future forecasts up to 2050: a systematic analysis for the global burden of disease study 2021
Source: Front Public Health. 2025 Jul 9;13:1612300. doi: 10.3389/fpubh.2025.1612300 (PMC12283729; doi:10.3389/fpubh.2025.1612300)
Supplement: Supplementary file 1 [file Table_1.docx]

| **Supplementary Table S1** The global disease burden of chronic kidney disease attributed to high body mass index in 204 countries and territories | | | | | |
| --- | --- | --- | --- | --- | --- |
| location | 1990 | | 2021 | | EAPC_CI |
|  | Number | ASR | Number | ASR |  |
| **Deaths** |  |  |  |  |  |
| Afghanistan | 627(311-1068) | 9.405(4.712-16.318) | 1219(502-2329) | 13.937(5.769-27.225) | 1.38 (1.19 to 1.58) |
| Albania | 60(33-95) | 3.428(1.904-5.448) | 159(85-256) | 3.826(2.024-6.11) | 0.68 (0.42 to 0.93) |
| Algeria | 531(262-1013) | 6.283(3.057-11.807) | 3542(1891-5422) | 13.293(7.129-20.682) | 2.91 (2.65 to 3.17) |
| American Samoa | 2(1-3) | 10.464(4.493-18.683) | 12(6-20) | 29.532(13.177-48.505) | 3.55 (3.29 to 3.82) |
| Andorra | 1(1-3) | 3.377(1.63-5.857) | 5(2-9) | 2.89(1.357-4.946) | -0.11 (-0.34 to 0.12) |
| Angola | 103(44-190) | 3.068(1.309-5.692) | 549(243-1002) | 5.558(2.436-10.062) | 1.71 (1.54 to 1.88) |
| Antigua and Barbuda | 5(2-7) | 8.357(4.407-13.433) | 18(10-27) | 17.768(9.938-27.279) | 3.11 (2.84 to 3.39) |
| Argentina | 2779(1487-4044) | 9.207(4.924-13.516) | 5963(3349-8607) | 10.263(5.774-14.833) | 0.59 (0.23 to 0.96) |
| Armenia | 5(2-8) | 0.189(0.087-0.296) | 124(61-196) | 2.855(1.408-4.542) | 8.7 (7.37 to 10.06) |
| Australia | 429(212-671) | 2.357(1.163-3.692) | 1930(1020-2895) | 3.564(1.904-5.31) | 2.04 (1.76 to 2.32) |
| Austria | 203(102-300) | 1.646(0.849-2.429) | 1154(605-1703) | 4.757(2.501-7.023) | 5.18 (4.5 to 5.87) |
| Azerbaijan | 67(33-112) | 1.349(0.66-2.204) | 286(146-480) | 2.944(1.476-4.999) | 2.94 (2.63 to 3.26) |
| Bahamas | 12(7-18) | 8.137(4.317-11.746) | 61(34-89) | 15.786(8.59-23.312) | 2.79 (2.55 to 3.03) |
| Bahrain | 13(7-22) | 10.856(5.37-19.364) | 115(70-167) | 22.87(13.173-34.297) | 2.12 (1.75 to 2.49) |
| Bangladesh | 343(151-636) | 0.777(0.339-1.412) | 1689(826-3001) | 1.338(0.646-2.363) | 2.03 (1.85 to 2.21) |
| Barbados | 21(11-31) | 7.083(3.963-10.494) | 68(38-105) | 13.291(7.491-20.264) | 2.6 (2.28 to 2.92) |
| Belarus | 17(8-27) | 0.139(0.064-0.22) | 97(49-152) | 0.606(0.305-0.953) | 4.81 (3.92 to 5.71) |
| Belgium | 348(177-533) | 2.187(1.117-3.325) | 940(455-1419) | 2.936(1.444-4.413) | 1.59 (1.29 to 1.89) |
| Belize | 8(4-11) | 8.569(4.541-12.435) | 56(33-79) | 19.452(11.004-28.459) | 3.12 (2.61 to 3.63) |
| Benin | 81(36-134) | 4.414(1.914-7.364) | 323(147-537) | 7.041(3.19-11.783) | 1.43 (1.34 to 1.53) |
| Bermuda | 4(2-6) | 6.972(3.964-9.501) | 12(7-17) | 8.25(4.831-11.571) | 1.06 (0.75 to 1.37) |
| Bhutan | 6(3-11) | 2.783(1.251-4.891) | 23(11-41) | 3.977(1.836-7.124) | 1.22 (1.17 to 1.27) |
| Bolivia (Plurinational State of) | 297(144-471) | 10.309(4.953-16.756) | 1602(842-2532) | 19.462(9.835-30.718) | 2.23 (2.14 to 2.33) |
| Bosnia and Herzegovina | 86(45-139) | 2.376(1.237-3.826) | 212(104-349) | 3.313(1.619-5.464) | 1.02 (0.81 to 1.22) |
| Botswana | 13(6-24) | 2.895(1.26-5.319) | 89(43-148) | 7.684(3.62-12.82) | 3.61 (3.31 to 3.9) |
| Brazil | 4375(2404-6346) | 5.499(2.967-8.072) | 19702(11197-27199) | 8.068(4.548-11.201) | 1.26 (1.06 to 1.45) |
| Brunei Darussalam | 4(2-6) | 4.361(2.023-7.63) | 17(9-27) | 6.225(3.076-10.369) | 1.71 (1.49 to 1.93) |
| Bulgaria | 238(131-348) | 2.396(1.313-3.504) | 859(453-1316) | 6.002(3.22-9.166) | 3.79 (3.32 to 4.27) |
| Burkina Faso | 99(43-176) | 2.712(1.187-4.887) | 321(139-577) | 4.004(1.745-7.108) | 1.31 (1.23 to 1.39) |
| Burundi | 46(21-85) | 2.221(1.027-4.174) | 111(51-209) | 2.835(1.284-5.239) | 0.32 (0.14 to 0.5) |
| Cabo Verde | 5(2-8) | 2.214(1.005-3.598) | 30(13-51) | 6.85(3.004-12.016) | 3.46 (3.2 to 3.71) |
| Cambodia | 52(22-97) | 1.273(0.542-2.378) | 206(82-373) | 1.849(0.745-3.341) | 1.12 (0.87 to 1.37) |
| Cameroon | 441(203-698) | 11.961(5.365-19.331) | 1869(942-3212) | 18.118(8.99-30.701) | 0.79 (0.55 to 1.04) |
| Canada | 743(375-1094) | 2.341(1.182-3.446) | 2891(1398-4368) | 3.466(1.687-5.209) | 1.62 (1.38 to 1.86) |
| Central African Republic | 45(21-77) | 4.425(1.992-7.672) | 142(66-254) | 7.12(3.349-13.119) | 1.56 (1.51 to 1.62) |
| Chad | 77(34-134) | 3.078(1.334-5.4) | 217(102-393) | 4.429(2.036-7.863) | 1.01 (0.89 to 1.14) |
| Chile | 455(251-653) | 5.057(2.789-7.306) | 1956(1030-2949) | 7.401(3.891-11.141) | 1.61 (1.15 to 2.07) |
| China | 11598(5254-22211) | 1.804(0.82-3.424) | 54112(27229-91108) | 2.83(1.405-4.731) | 1.41 (1.31 to 1.51) |
| Colombia | 667(331-1033) | 4.27(2.101-6.704) | 2766(1420-4337) | 4.911(2.521-7.739) | 0.8 (0.63 to 0.97) |
| Comoros | 5(2-10) | 3.222(1.411-5.964) | 28(13-52) | 6.645(3.028-12.354) | 2.21 (2.1 to 2.31) |
| Congo | 63(26-108) | 6.551(2.701-11.549) | 245(103-425) | 10.291(4.227-17.919) | 1.21 (1.07 to 1.35) |
| Cook Islands | 1(0-1) | 5.678(2.557-9.559) | 2(1-4) | 8.814(4.278-14.124) | 1.47 (1.37 to 1.57) |
| Costa Rica | 73(38-109) | 4.367(2.29-6.574) | 553(289-853) | 9.902(5.217-15.24) | 2.77 (2.33 to 3.22) |
| C么te d'Ivoire | 181(84-303) | 5.593(2.598-9.315) | 815(400-1388) | 8.542(4.038-14.106) | 1.85 (1.53 to 2.18) |
| Croatia | 150(81-225) | 2.798(1.515-4.191) | 490(252-746) | 4.897(2.518-7.447) | 4.17 (3.81 to 4.53) |
| Cuba | 210(109-319) | 2.122(1.112-3.212) | 1220(687-1850) | 6.082(3.442-9.136) | -0.81 (-1.07 to -0.55) |
| Cyprus | 33(15-59) | 7.334(3.354-13.066) | 91(42-160) | 5.957(2.829-9.983) | -0.29 (-0.49 to -0.08) |
| Czechia | 380(206-556) | 2.774(1.534-4.068) | 578(306-899) | 2.472(1.304-3.822) | 1.22 (1.11 to 1.34) |
| Democratic People's Republic of Korea | 246(104-451) | 2.014(0.845-3.771) | 884(397-1614) | 3.017(1.36-5.5) | 1.45 (1.38 to 1.52) |
| Democratic Republic of the Congo | 708(363-1197) | 5.658(2.842-9.665) | 2880(1367-5054) | 9.732(4.651-17.055) | 1.59 (1.44 to 1.75) |
| Denmark | 96(48-145) | 1.095(0.549-1.643) | 506(252-772) | 3.546(1.772-5.414) | 3.94 (3.69 to 4.19) |
| Djibouti | 2(1-4) | 1.766(0.778-3.532) | 19(9-37) | 3.954(1.72-7.503) | 2.61 (2.5 to 2.71) |
| Dominica | 7(4-10) | 12.109(6.48-18.038) | 19(11-28) | 23.98(13.541-35.315) | 2.47 (2.38 to 2.56) |
| Dominican Republic | 160(83-246) | 4.827(2.462-7.462) | 939(485-1441) | 9.484(4.886-14.633) | 3.04 (2.77 to 3.31) |
| Ecuador | 375(209-543) | 7.819(4.277-11.576) | 2833(1552-4440) | 18.393(10.024-28.346) | 2.92 (2.02 to 3.82) |
| Egypt | 2922(1591-4824) | 15.569(8.313-26.134) | 14166(7865-20836) | 30.743(16.229-45.357) | 2.51 (2.41 to 2.62) |
| El Salvador | 207(102-339) | 7.097(3.472-11.741) | 1560(828-2419) | 24.518(12.942-38.094) | 4.09 (3.58 to 4.61) |
| Equatorial Guinea | 11(5-19) | 6.502(3.172-11.176) | 70(30-125) | 16.467(7.066-29.433) | 3.42 (3.02 to 3.83) |
| Eritrea | 12(5-22) | 1.264(0.533-2.413) | 60(25-121) | 2.726(1.119-5.428) | 2.61 (2.54 to 2.67) |
| Estonia | 25(12-40) | 1.258(0.617-2.034) | 182(96-265) | 5.593(2.93-8.219) | 4.54 (3.92 to 5.16) |
| Eswatini | 20(10-33) | 8.881(4.37-14.592) | 89(44-147) | 19.603(9.914-31.447) | 2.93 (2.25 to 3.61) |
| Ethiopia | 689(306-1286) | 4.09(1.839-7.579) | 1519(673-2717) | 4.069(1.764-7.4) | -0.43 (-0.6 to -0.26) |
| Fiji | 23(11-40) | 7.541(3.496-13.685) | 117(54-187) | 19.11(8.899-30.38) | 2.54 (2.12 to 2.96) |
| Finland | 60(30-92) | 0.842(0.417-1.281) | 246(115-383) | 1.427(0.676-2.225) | 2.24 (2.02 to 2.47) |
| France | 1704(867-2665) | 1.896(0.981-2.934) | 5069(2497-7485) | 2.391(1.181-3.522) | 1.24 (1 to 1.48) |
| Gabon | 51(24-79) | 10.093(4.764-15.817) | 196(84-320) | 23.259(9.279-39.489) | 2.63 (2.41 to 2.85) |
| Gambia | 15(7-24) | 4.856(2.213-8.117) | 77(38-133) | 8.697(4.218-15.487) | 1.69 (1.56 to 1.81) |
| Georgia | 42(20-67) | 0.684(0.331-1.086) | 176(80-300) | 2.936(1.334-4.927) | 5.04 (4.36 to 5.72) |
| Germany | 3010(1447-4739) | 2.234(1.076-3.503) | 11448(4984-18852) | 4.406(1.947-7.264) | 3.46 (2.98 to 3.94) |
| Ghana | 186(85-317) | 3.695(1.658-6.488) | 1522(783-2530) | 11.433(5.634-18.554) | 3.94 (3.85 to 4.03) |
| Greece | 831(442-1235) | 5.882(3.086-8.733) | 2430(1223-3633) | 7.224(3.705-10.666) | 0.09 (-0.89 to 1.08) |
| Greenland | 1(0-1) | 4.1(1.96-6.232) | 2(1-4) | 4.746(2.071-7.855) | 0.95 (0.76 to 1.15) |
| Grenada | 6(3-10) | 8.376(4.344-13.016) | 22(12-34) | 19.802(10.881-31.467) | 3.32 (3.11 to 3.53) |
| Guam | 4(2-7) | 7.611(3.882-11.75) | 18(9-28) | 8.147(4.08-12.872) | 1.1 (0.7 to 1.49) |
| Guatemala | 228(117-336) | 8.356(4.247-12.282) | 1634(849-2621) | 15.597(7.956-24.853) | 2.88 (2.46 to 3.3) |
| Guinea | 114(54-197) | 3.836(1.755-6.612) | 290(131-510) | 5.676(2.525-10.086) | 1.3 (1.24 to 1.36) |
| Guinea-Bissau | 20(9-35) | 5.661(2.669-9.977) | 48(22-87) | 7.745(3.436-13.739) | 0.9 (0.83 to 0.96) |
| Guyana | 31(17-48) | 8.53(4.765-13.54) | 130(72-205) | 21.102(11.53-33.239) | 4.09 (3.71 to 4.47) |
| Haiti | 93(41-176) | 3.037(1.316-5.878) | 372(145-808) | 5.233(2.044-11.637) | 2.13 (2 to 2.26) |
| Honduras | 52(26-86) | 2.765(1.343-4.578) | 415(196-687) | 7.176(3.405-11.888) | 3.39 (3.14 to 3.65) |
| Hungary | 256(139-387) | 1.833(1.002-2.793) | 732(360-1153) | 3.39(1.679-5.347) | 3.03 (2.71 to 3.36) |
| Iceland | 3(2-5) | 1.091(0.584-1.629) | 14(7-22) | 2.006(0.996-3.019) | 2.5 (2.31 to 2.7) |
| India | 3839(1918-6552) | 0.91(0.459-1.559) | 24249(12246-39900) | 2.177(1.088-3.617) | 2.85 (2.75 to 2.95) |
| Indonesia | 1250(573-2175) | 1.332(0.623-2.308) | 6952(3105-11496) | 3(1.372-4.962) | 2.82 (2.68 to 2.96) |
| Iran (Islamic Republic of) | 788(392-1305) | 3.834(1.848-6.553) | 4906(2531-7439) | 7.235(3.672-11.041) | 2.05 (1.91 to 2.18) |
| Iraq | 1022(563-1562) | 13.601(7.392-21.051) | 3284(1590-5417) | 17.561(8.406-28.74) | 0.44 (0.23 to 0.64) |
| Ireland | 80(39-123) | 2.124(1.041-3.208) | 235(120-357) | 2.701(1.38-4.096) | 1.48 (1.27 to 1.69) |
| Israel | 282(143-426) | 6.473(3.366-9.856) | 857(403-1347) | 5.958(2.784-9.393) | 0.36 (-0.14 to 0.86) |
| Italy | 1988(1019-3029) | 2.278(1.156-3.48) | 6243(3047-9652) | 2.959(1.444-4.551) | 0.81 (0.67 to 0.95) |
| Jamaica | 100(47-163) | 5.472(2.577-8.87) | 341(171-536) | 10.605(5.33-16.631) | 1.61 (0.97 to 2.25) |
| Japan | 3070(1480-5016) | 2(0.96-3.296) | 10715(5087-17613) | 1.905(0.925-3.087) | -0.26 (-0.38 to -0.14) |
| Jordan | 120(64-182) | 11.238(5.697-17.455) | 846(495-1220) | 15.541(8.682-22.56) | 0.88 (0.56 to 1.2) |
| Kazakhstan | 139(69-224) | 1.068(0.532-1.716) | 524(254-873) | 3.233(1.553-5.378) | 3.24 (2.86 to 3.62) |
| Kenya | 175(81-317) | 2.466(1.155-4.502) | 1259(611-2071) | 6.616(3.128-11.121) | 3.51 (3.41 to 3.61) |
| Kiribati | 2(1-4) | 7.259(3.174-12.673) | 9(4-16) | 14.118(5.659-27.198) | 2 (1.73 to 2.27) |
| Kuwait | 48(27-72) | 9.7(4.918-15.299) | 180(102-261) | 7.998(4.389-11.675) | -0.5 (-0.75 to -0.24) |
| Kyrgyzstan | 22(11-37) | 0.706(0.361-1.173) | 119(62-187) | 2.521(1.285-3.956) | 2.6 (1.67 to 3.53) |
| Lao People's Democratic Republic | 59(27-108) | 3.13(1.418-5.717) | 193(75-368) | 4.524(1.758-8.503) | 1.22 (1.19 to 1.24) |
| Latvia | 20(9-31) | 0.566(0.269-0.913) | 106(55-163) | 2.395(1.222-3.725) | 4.93 (4.45 to 5.42) |
| Lebanon | 171(86-283) | 9.253(4.613-15.202) | 860(447-1339) | 13.095(6.908-20.288) | 1.37 (1.2 to 1.55) |
| Lesotho | 22(11-39) | 3.061(1.389-5.331) | 109(48-191) | 11.933(5.13-20.856) | 5.55 (4.96 to 6.14) |
| Liberia | 83(43-137) | 7.996(4.011-12.976) | 240(117-411) | 12.336(5.889-21.593) | 1.67 (1.35 to 1.99) |
| Libya | 122(59-204) | 7.019(3.355-11.964) | 863(407-1405) | 19.005(8.85-30.93) | 4.04 (3.78 to 4.29) |
| Lithuania | 20(10-32) | 0.453(0.22-0.722) | 108(58-162) | 1.726(0.922-2.659) | 3.21 (2.72 to 3.7) |
| Luxembourg | 15(8-22) | 2.901(1.484-4.309) | 48(21-79) | 3.752(1.687-6.166) | 1.29 (1.01 to 1.58) |
| Madagascar | 75(33-141) | 1.777(0.777-3.389) | 282(116-513) | 3.337(1.354-5.975) | 2.04 (1.91 to 2.17) |
| Malawi | 107(46-199) | 3.422(1.505-6.495) | 413(196-720) | 6.693(3.085-11.805) | 2.02 (1.81 to 2.23) |
| Malaysia | 378(197-603) | 4.251(2.239-6.763) | 2089(1095-3213) | 7.912(4.114-12.189) | 1.78 (1.58 to 1.99) |
| Maldives | 5(2-8) | 5.271(2.317-9.665) | 15(7-26) | 4.556(1.989-8.11) | -0.79 (-1.01 to -0.57) |
| Mali | 165(81-283) | 4.95(2.406-8.387) | 449(232-768) | 5.978(2.898-10.314) | 0.68 (0.52 to 0.83) |
| Malta | 10(5-16) | 2.771(1.392-4.279) | 41(19-67) | 3.514(1.624-5.733) | 0.86 (0.54 to 1.19) |
| Marshall Islands | 1(0-2) | 7.552(2.934-15.932) | 5(1-12) | 15.298(3.453-43.501) | 2.24 (2.01 to 2.47) |
| Mauritania | 81(40-131) | 9.639(4.603-15.542) | 241(118-402) | 13.584(6.632-22.669) | 0.75 (0.58 to 0.93) |
| Mauritius | 50(24-81) | 7.223(3.417-11.972) | 380(189-607) | 20.915(10.291-33.265) | 3.7 (3.27 to 4.14) |
| Mexico | 3595(1870-5598) | 9.921(5.145-15.895) | 24462(13956-35479) | 19.852(11.274-28.913) | 2.84 (2.15 to 3.53) |
| Micronesia (Federated States of) | 4(2-7) | 8.814(3.917-17.191) | 12(5-21) | 18(7.094-32.952) | 2.32 (1.93 to 2.7) |
| Monaco | 2(1-3) | 1.955(1.028-2.979) | 5(2-7) | 3.507(1.814-5.231) | 2.11 (1.83 to 2.4) |
| Mongolia | 31(16-50) | 3.031(1.547-4.868) | 74(40-122) | 3.461(1.847-5.825) | 0.02 (-0.13 to 0.18) |
| Montenegro | 27(15-40) | 4.541(2.482-6.847) | 67(36-102) | 7.677(3.934-11.628) | 1.88 (1.6 to 2.16) |
| Morocco | 772(374-1428) | 5.981(2.9-11.32) | 3845(1984-6260) | 12.642(6.474-20.621) | 2.7 (2.58 to 2.83) |
| Mozambique | 83(34-150) | 1.683(0.705-3.156) | 374(162-699) | 3.941(1.693-7.395) | 3.22 (3.08 to 3.36) |
| Myanmar | 485(202-854) | 2.171(0.939-3.815) | 1315(596-2336) | 2.821(1.282-4.927) | 0.64 (0.57 to 0.72) |
| Namibia | 19(9-33) | 3.594(1.629-6.158) | 89(45-147) | 7.986(4.063-13.082) | 2.42 (2.07 to 2.77) |
| Nauru | 0(0-1) | 10.602(4.69-19.475) | 1(1-2) | 22.049(9.541-39.943) | 2.21 (2.08 to 2.33) |
| Nepal | 85(38-150) | 0.928(0.401-1.664) | 498(234-905) | 2.199(1.067-4.012) | 3.13 (2.8 to 3.46) |
| Netherlands | 331(158-519) | 1.633(0.782-2.564) | 1386(701-2138) | 3.357(1.7-5.175) | 2.58 (2.2 to 2.96) |
| New Zealand | 84(45-126) | 2.24(1.199-3.333) | 365(201-525) | 3.988(2.217-5.725) | 2.16 (1.71 to 2.6) |
| Nicaragua | 104(49-163) | 7.138(3.337-11.484) | 849(429-1375) | 17.899(8.956-29.022) | 3.6 (3.17 to 4.03) |
| Niger | 69(32-121) | 2.974(1.356-5.148) | 230(102-417) | 3.321(1.472-5.941) | 0.26 (0.15 to 0.37) |
| Nigeria | 1503(736-2520) | 4.171(1.989-7.012) | 5378(2703-8724) | 7.531(3.717-12.053) | 1.75 (1.66 to 1.84) |
| Niue | 0(0-0) | 8.156(3.636-14.518) | 0(0-1) | 20.512(7.765-37.975) | 2.99 (2.79 to 3.19) |
| North Macedonia | 49(26-78) | 2.879(1.498-4.523) | 126(61-208) | 4.54(2.26-7.437) | 1.46 (1.11 to 1.82) |
| Northern Mariana Islands | 2(1-3) | 12.337(5.45-21.088) | 9(4-15) | 22.134(9.617-36.874) | 2.06 (1.87 to 2.25) |
| Norway | 71(37-110) | 0.922(0.472-1.42) | 243(125-379) | 1.929(0.987-3.001) | 2.72 (2.38 to 3.06) |
| Oman | 29(13-48) | 4.904(2.164-8.411) | 259(146-392) | 17.428(9.751-26.445) | 5 (4.65 to 5.35) |
| Pakistan | 1307(646-2181) | 2.499(1.226-4.208) | 6252(2948-10489) | 5.472(2.624-9.108) | 2.5 (2.24 to 2.76) |
| Palau | 1(0-1) | 9.12(4.26-15.961) | 3(2-6) | 19.005(8.6-32.328) | 2.57 (2.36 to 2.78) |
| Palestine | 98(50-166) | 13.461(6.815-23.073) | 320(172-486) | 16.991(8.695-26.084) | 0.7 (0.46 to 0.93) |
| Panama | 59(31-86) | 4.262(2.243-6.194) | 532(284-791) | 11.786(6.283-17.556) | 3.62 (3.15 to 4.09) |
| Papua New Guinea | 24(10-49) | 1.406(0.57-2.803) | 88(35-176) | 1.771(0.702-3.64) | 0.64 (0.57 to 0.72) |
| Paraguay | 123(66-181) | 5.924(3.189-8.871) | 587(296-909) | 10.614(5.301-16.486) | 2.18 (2.05 to 2.31) |
| Peru | 868(443-1288) | 7.822(3.96-11.677) | 3987(2045-6223) | 11.937(6.093-18.676) | 1.13 (0.92 to 1.34) |
| Philippines | 674(300-1161) | 2.683(1.186-4.614) | 4725(1969-8254) | 6.03(2.511-10.556) | 3.11 (2.92 to 3.31) |
| Poland | 1550(812-2307) | 3.698(1.945-5.528) | 1699(933-2649) | 2.214(1.21-3.454) | -1.89 (-2.5 to -1.27) |
| Portugal | 420(219-659) | 3.376(1.763-5.322) | 1526(742-2398) | 4.587(2.26-7.202) | 0.96 (0.46 to 1.45) |
| Puerto Rico | 402(226-608) | 11.808(6.682-17.949) | 1187(678-1821) | 14.661(8.668-21.857) | 1.41 (0.99 to 1.83) |
| Qatar | 8(4-15) | 12.656(5.481-23.418) | 92(57-134) | 19.813(11.595-28.62) | 1.01 (0.35 to 1.68) |
| Republic of Korea | 527(235-885) | 2.533(1.094-4.302) | 2063(953-3472) | 2.267(1.052-3.834) | -0.18 (-0.36 to 0.01) |
| Republic of Moldova | 16(8-25) | 0.402(0.199-0.628) | 66(36-103) | 1.109(0.595-1.734) | 2.78 (2.3 to 3.27) |
| Romania | 732(394-1142) | 2.916(1.544-4.542) | 1258(725-1851) | 3.201(1.841-4.704) | 0.63 (0.15 to 1.12) |
| Russian Federation | 1644(852-2558) | 0.935(0.483-1.445) | 4603(2479-6740) | 1.903(1.021-2.785) | 1.92 (1.45 to 2.4) |
| Rwanda | 72(30-136) | 2.955(1.27-5.57) | 196(79-372) | 3.844(1.563-7.341) | 0 (-0.37 to 0.38) |
| Saint Kitts and Nevis | 4(2-7) | 12.523(6.621-19.985) | 14(8-21) | 23.068(12.559-35.016) | 2.94 (2.63 to 3.25) |
| Saint Lucia | 7(4-11) | 8.743(4.394-13.914) | 32(17-50) | 13.663(7.091-21.526) | 1.76 (1.47 to 2.06) |
| Saint Vincent and the Grenadines | 4(2-6) | 5.419(2.675-8.734) | 17(9-27) | 12.518(6.464-19.946) | 3.45 (3.1 to 3.79) |
| Samoa | 7(3-12) | 8.969(3.899-16.184) | 22(10-35) | 16.557(7.461-26.914) | 1.99 (1.9 to 2.07) |
| San Marino | 1(0-1) | 1.658(0.848-2.548) | 2(1-3) | 1.35(0.612-2.271) | 0.62 (0.18 to 1.05) |
| Sao Tome and Principe | 5(3-9) | 9.287(4.555-14.995) | 18(9-29) | 19.662(9.162-31.439) | 2.46 (2.4 to 2.52) |
| Saudi Arabia | 667(338-1108) | 12.893(6.273-21.606) | 5336(3151-7851) | 35.617(19.801-53.057) | 3.03 (2.82 to 3.25) |
| Senegal | 182(90-298) | 6.445(3.24-10.579) | 593(281-1003) | 8.881(4.173-14.852) | 0.83 (0.71 to 0.96) |
| Serbia | 419(221-679) | 4.649(2.432-7.667) | 996(531-1546) | 5.73(3.062-8.906) | 0.63 (0.5 to 0.76) |
| Seychelles | 3(2-6) | 6.207(3.177-9.884) | 16(8-25) | 14.628(6.899-23.829) | 2.97 (2.73 to 3.22) |
| Sierra Leone | 67(33-115) | 3.722(1.853-6.431) | 163(78-285) | 4.975(2.39-8.573) | 0.88 (0.84 to 0.91) |
| Singapore | 35(17-57) | 1.833(0.87-3.078) | 184(84-304) | 2.22(1.012-3.683) | 2.04 (1.46 to 2.62) |
| Slovakia | 210(117-316) | 3.558(1.983-5.357) | 330(183-489) | 3.431(1.914-5.087) | -0.04 (-0.1 to 0.01) |
| Slovenia | 47(25-72) | 1.947(1.034-2.988) | 128(68-197) | 2.35(1.251-3.66) | 1.41 (1.1 to 1.71) |
| Solomon Islands | 5(2-10) | 3.735(1.308-8.141) | 14(5-28) | 4.236(1.495-8.848) | 0.16 (-0.04 to 0.36) |
| Somalia | 63(29-124) | 3.099(1.392-6.036) | 258(112-488) | 4.89(2.067-9.301) | 1.61 (1.52 to 1.69) |
| South Africa | 822(409-1334) | 4.283(2.121-7.051) | 4571(2380-6980) | 11.376(5.836-17.636) | 3.07 (2.61 to 3.53) |
| South Sudan | 52(23-110) | 2.338(1.02-4.828) | 147(61-285) | 4.547(1.875-8.669) | 2.1 (1.88 to 2.32) |
| Spain | 1913(948-3013) | 3.651(1.817-5.73) | 4891(2225-7850) | 3.402(1.587-5.424) | -0.04 (-0.14 to 0.06) |
| Sri Lanka | 231(100-412) | 2.445(1.021-4.36) | 895(359-1740) | 3.527(1.404-6.829) | 1.19 (1 to 1.38) |
| Sudan | 408(200-738) | 4.867(2.325-9.076) | 1802(856-3048) | 10.495(4.92-17.556) | 2.58 (2.37 to 2.79) |
| Suriname | 16(9-26) | 6.415(3.33-10.405) | 79(39-132) | 12.632(6.212-21.303) | 2.55 (2.35 to 2.75) |
| Sweden | 157(78-242) | 0.912(0.453-1.415) | 733(362-1177) | 2.51(1.246-4.022) | 3.72 (3.55 to 3.88) |
| Switzerland | 201(92-322) | 1.758(0.82-2.801) | 649(274-1116) | 2.486(1.056-4.295) | 2.15 (1.8 to 2.5) |
| Syrian Arab Republic | 513(274-830) | 11.378(5.941-18.355) | 2104(1086-3298) | 20.373(10.288-31.801) | 1.51 (1.25 to 1.77) |
| Taiwan | 611(276-1052) | 5.209(2.382-9.174) | 2537(1163-4423) | 5.607(2.567-9.763) | 0.58 (0.26 to 0.9) |
| Tajikistan | 9(4-16) | 0.332(0.155-0.567) | 33(15-57) | 0.581(0.258-0.981) | 1.34 (0.92 to 1.77) |
| Thailand | 746(325-1324) | 2.366(1.038-4.169) | 6240(2547-11055) | 5.708(2.324-10.129) | 2.54 (2.32 to 2.76) |
| Timor-Leste | 3(1-6) | 1.21(0.478-2.384) | 15(6-30) | 1.899(0.786-3.844) | 1.76 (1.42 to 2.1) |
| Togo | 43(21-72) | 4.316(2.037-7.246) | 220(106-376) | 7.622(3.636-13.228) | 1.68 (1.61 to 1.75) |
| Tokelau | 0(0-0) | 5.881(2.682-11.465) | 0(0-0) | 11.906(5.174-20.708) | 2.35 (2.18 to 2.53) |
| Tonga | 2(1-4) | 4.092(1.667-7.344) | 7(3-11) | 8.426(3.517-13.945) | 2.24 (2.01 to 2.46) |
| Trinidad and Tobago | 53(28-80) | 6.899(3.701-10.563) | 304(160-481) | 15.818(8.334-25.073) | 3.39 (3.02 to 3.77) |
| Tunisia | 172(82-296) | 4.019(1.904-6.987) | 1203(578-2042) | 10.12(4.909-17.522) | 3.1 (3.03 to 3.16) |
| Turkey | 2731(1438-4313) | 9.424(4.903-14.83) | 10281(5592-15680) | 12.111(6.539-18.48) | 1.24 (0.78 to 1.71) |
| Turkmenistan | 36(19-57) | 1.784(0.946-2.828) | 192(97-330) | 4.76(2.364-8.179) | 2.85 (2.27 to 3.43) |
| Tuvalu | 0(0-1) | 5.132(1.983-10.484) | 1(0-2) | 11.104(4.136-22.818) | 2.58 (2.47 to 2.68) |
| Uganda | 127(54-237) | 2.259(0.992-4.184) | 558(245-1034) | 4.395(1.978-8.297) | 1.81 (1.64 to 1.98) |
| Ukraine | 16(8-25) | 0.024(0.012-0.038) | 409(200-684) | 0.561(0.275-0.958) | 14.26 (12.24 to 16.31) |
| United Arab Emirates | 23(12-39) | 6.294(3.059-11.167) | 313(169-449) | 17.941(8.317-26.523) | 5.92 (5.12 to 6.73) |
| United Kingdom | 998(450-1521) | 1.049(0.47-1.598) | 2486(1093-3997) | 1.548(0.682-2.472) | 2.07 (1.75 to 2.39) |
| United Republic of Tanzania | 361(177-620) | 3.938(1.829-6.674) | 1637(835-2785) | 7.454(3.795-12.471) | 2.04 (1.99 to 2.09) |
| United States of America | 10066(5407-14042) | 3.042(1.647-4.23) | 62324(33545-89159) | 9.982(5.472-14.148) | 0.94 (0.66 to 1.21) |
| United States Virgin Islands | 6(3-9) | 7.896(4.383-11.838) | 16(8-25) | 9.44(4.929-14.495) | 4.27 (4.04 to 4.49) |
| Uruguay | 145(70-218) | 3.731(1.81-5.584) | 353(172-553) | 5.367(2.624-8.34) | 1.51 (1.25 to 1.76) |
| Uzbekistan | 111(47-219) | 1.036(0.432-2.063) | 901(441-1505) | 3.61(1.759-6.083) | 3 (2.16 to 3.85) |
| Vanuatu | 3(1-5) | 5.178(2.331-10.16) | 13(5-24) | 8.169(3.42-15.579) | 1.41 (1.35 to 1.46) |
| Venezuela (Bolivarian Republic of) | 447(240-663) | 4.966(2.639-7.363) | 4232(2202-6691) | 14.5(7.49-23.064) | 3.19 (2.74 to 3.65) |
| Viet Nam | 384(163-716) | 1.047(0.439-1.961) | 1943(789-3588) | 2.176(0.862-3.99) | 2.76 (2.56 to 2.95) |
| Yemen | 124(59-240) | 2.969(1.451-5.786) | 618(297-1117) | 5.312(2.522-9.797) | 1.79 (1.56 to 2.03) |
| Zambia | 102(47-181) | 4.169(1.949-7.58) | 535(253-971) | 8.773(4.06-15.817) | 2.06 (1.87 to 2.25) |
| Zimbabwe | 97(43-175) | 2.886(1.248-5.204) | 521(243-898) | 8.872(4.041-15.448) | 4.19 (3.61 to 4.78) |
| **DALYs** |  |  |  |  |  |
| Afghanistan | 18210(9208-30928) | 251.759(127.753-426.852) | 38005(16015-70229) | 349.198(147.42-672.033) | 1.15 (0.99 to 1.31) |
| Albania | 1773(987-2737) | 88.535(49.801-135.775) | 3948(2218-5875) | 95.011(53.109-142.542) | 0.44 (0.25 to 0.62) |
| Algeria | 14503(7405-26270) | 129.1(64.657-236.711) | 80765(44360-121727) | 245.262(133.864-367.67) | 2.32 (2.12 to 2.52) |
| American Samoa | 62(30-101) | 264.649(120.492-442.234) | 321(156-487) | 668.9(321.389-1029.317) | 3.19 (2.95 to 3.43) |
| Andorra | 38(20-62) | 73.29(38.016-116.975) | 110(57-174) | 67.422(35.337-106.494) | -0.03 (-0.2 to 0.13) |
| Angola | 3450(1490-6378) | 80.605(35.098-149.517) | 18177(7999-32143) | 138.358(60.692-246.078) | 1.55 (1.4 to 1.7) |
| Antigua and Barbuda | 110(59-170) | 212.739(115.513-327.199) | 429(248-624) | 399.669(228.973-585.873) | 2.67 (2.42 to 2.93) |
| Argentina | 60441(33276-85796) | 190.447(103.96-270.982) | 117059(66099-165983) | 208.127(117.733-296.095) | 0.53 (0.21 to 0.84) |
| Armenia | 1157(613-1806) | 42.359(22.532-66.478) | 4565(2417-6939) | 107.261(56.725-162.377) | 3.25 (2.92 to 3.59) |
| Australia | 10062(5229-15310) | 52.943(27.581-80.697) | 35023(19627-49395) | 73.268(41.612-102.96) | 1.45 (1.26 to 1.64) |
| Austria | 5624(2868-8396) | 47.519(24.392-70.357) | 18163(9611-26300) | 86.803(46.084-125.733) | 2.99 (2.59 to 3.39) |
| Azerbaijan | 4121(2252-6420) | 78.369(42.834-120.743) | 13588(7707-21130) | 125.4(68.675-197.576) | 1.67 (1.52 to 1.83) |
| Bahamas | 385(218-531) | 227.905(126.542-320.318) | 1717(1000-2413) | 404.76(233.52-573.789) | 2.44 (2.24 to 2.64) |
| Bahrain | 398(214-671) | 225.692(117.44-392.945) | 3185(1944-4494) | 410.008(243.392-604.206) | 1.53 (1.23 to 1.83) |
| Bangladesh | 12073(5066-22298) | 23.579(10.134-43.473) | 60310(29134-102733) | 42.097(20.311-72.261) | 2.39 (2.21 to 2.58) |
| Barbados | 493(282-718) | 182.209(105.775-261.377) | 1509(862-2261) | 304.953(176.395-449.299) | 2.18 (1.92 to 2.44) |
| Belarus | 2980(1446-4725) | 23.701(11.469-37.651) | 6526(3601-9780) | 42.012(23.07-63.259) | 1.87 (1.59 to 2.14) |
| Belgium | 8731(4419-12950) | 56.662(28.608-83.555) | 17902(9002-26260) | 69.908(36.152-102.232) | 1.17 (0.98 to 1.37) |
| Belize | 211(119-289) | 219.333(123.043-302.313) | 1533(954-2075) | 476.823(287.195-657.707) | 2.95 (2.51 to 3.4) |
| Benin | 2257(1010-3729) | 109.888(49.143-181.753) | 9691(4473-16179) | 170.523(78.861-282.52) | 1.33 (1.23 to 1.43) |
| Bermuda | 105(62-140) | 167.933(98.431-224.354) | 244(147-334) | 184.563(115.272-250.487) | 0.76 (0.49 to 1.03) |
| Bhutan | 237(112-397) | 85.619(41.047-145.257) | 719(333-1221) | 113.043(52.257-192.932) | 0.91 (0.84 to 0.97) |
| Bolivia (Plurinational State of) | 7959(3841-12420) | 243.719(118.812-380.316) | 38771(21299-59926) | 424.284(229.399-660.305) | 1.88 (1.79 to 1.97) |
| Bosnia and Herzegovina | 2901(1536-4450) | 71.295(37.875-109.408) | 5372(2684-8384) | 88.228(43.933-138.398) | 0.66 (0.49 to 0.83) |
| Botswana | 432(198-755) | 77.269(35.171-136.743) | 2667(1279-4461) | 180.135(85.657-294.211) | 3.04 (2.8 to 3.27) |
| Brazil | 137857(76545-196760) | 148.599(81.201-215.293) | 494253(286017-670611) | 196.576(113.178-268.03) | 0.8 (0.61 to 1) |
| Brunei Darussalam | 110(52-186) | 100.29(46.708-169.178) | 522(269-818) | 144.516(72.516-227.226) | 1.53 (1.39 to 1.66) |
| Bulgaria | 8594(4768-12350) | 76.257(42.282-110.847) | 21023(11483-31991) | 159.734(86.521-242.891) | 2.97 (2.63 to 3.3) |
| Burkina Faso | 2882(1230-5118) | 65.427(28.475-116.87) | 9420(4167-16779) | 94.722(40.68-167.637) | 1.24 (1.15 to 1.33) |
| Burundi | 1225(552-2273) | 51.918(23.669-95.705) | 3120(1456-5920) | 62.799(28.553-116.857) | 0.13 (-0.05 to 0.32) |
| Cabo Verde | 144(68-235) | 64.563(30.501-103.942) | 755(366-1268) | 159.879(76.168-271.176) | 2.78 (2.62 to 2.93) |
| Cambodia | 1664(702-3020) | 34.617(14.646-63.78) | 6209(2485-11700) | 47.648(19.335-87.095) | 0.93 (0.71 to 1.14) |
| Cameroon | 12125(5951-19142) | 271.054(128.379-428.959) | 55674(28263-93590) | 414.353(210.713-690.39) | 0.82 (0.54 to 1.09) |
| Canada | 16452(8519-23514) | 50.963(26.33-72.638) | 51604(25800-74930) | 70.55(35.91-100.377) | 1.39 (1.19 to 1.59) |
| Central African Republic | 1531(691-2599) | 119.645(54.128-203.859) | 5087(2341-8963) | 191.894(90.393-337.19) | 1.53 (1.47 to 1.58) |
| Chad | 2096(942-3520) | 75.231(34.405-125.675) | 6565(3077-11565) | 107.44(50.507-190.405) | 0.98 (0.84 to 1.11) |
| Chile | 11600(6531-16765) | 117.286(66.115-169.327) | 37523(20610-54668) | 145.381(80.101-211.69) | 1.01 (0.59 to 1.42) |
| China | 349224(157171-671163) | 43.492(19.705-82.983) | 1385819(702691-2257375) | 66.968(33.815-109.54) | 1.44 (1.32 to 1.56) |
| Colombia | 19854(9925-30494) | 110.584(54.863-168.991) | 69548(35646-103957) | 124.727(63.951-186.931) | 0.62 (0.46 to 0.77) |
| Comoros | 149(65-268) | 74.127(32.646-132.911) | 722(315-1307) | 146.396(64.442-264.856) | 2.03 (1.91 to 2.16) |
| Congo | 1976(812-3331) | 172.35(70.856-287.811) | 8052(3613-14081) | 259.306(113.654-444.167) | 1.05 (0.91 to 1.19) |
| Cook Islands | 19(9-30) | 151.973(73.181-242.186) | 55(28-85) | 217.945(108.549-330.339) | 1.25 (1.17 to 1.32) |
| Costa Rica | 2267(1192-3385) | 126.578(67.065-190.022) | 14039(7724-20761) | 253.042(139.154-375.16) | 2.3 (1.94 to 2.66) |
| C么te d'Ivoire | 5995(2792-10102) | 135.931(64.093-224.656) | 26492(12715-45922) | 207.149(102.011-351.763) | 1.06 (0.85 to 1.27) |
| Croatia | 4459(2468-6620) | 76.758(42.465-114.724) | 9763(5117-14647) | 107.473(56.647-161.414) | 3.32 (3.02 to 3.62) |
| Cuba | 6800(3604-9962) | 66.502(35.284-97.73) | 29264(17039-42193) | 155.655(91.505-221.501) | -0.53 (-0.7 to -0.36) |
| Cyprus | 682(322-1153) | 114.516(54.001-194.277) | 1802(856-2983) | 100.459(48.58-164.698) | -0.67 (-0.79 to -0.54) |
| Czechia | 11493(6519-16515) | 85.389(48.653-122.856) | 14393(7795-21727) | 67.702(37.331-101.922) | 1.21 (1.09 to 1.32) |
| Democratic People's Republic of Korea | 7033(2963-12872) | 47.513(20.127-86.953) | 22043(9677-40675) | 69.848(30.655-128.196) | 1.31 (1.27 to 1.34) |
| Democratic Republic of the Congo | 22033(10993-36930) | 137.875(70.459-231.915) | 88969(42912-152590) | 230.877(110.991-394.066) | 1.5 (1.36 to 1.63) |
| Denmark | 3097(1529-4637) | 38.027(18.744-57.25) | 9300(4799-13951) | 73.398(38.534-109.805) | 2.08 (1.94 to 2.22) |
| Djibouti | 56(25-111) | 40.013(17.918-77.636) | 574(251-1076) | 86.776(38.459-162.948) | 2.5 (2.38 to 2.61) |
| Dominica | 164(89-237) | 284.105(154.746-409.1) | 457(264-657) | 550.831(316.242-794.712) | 2.42 (2.33 to 2.52) |
| Dominican Republic | 4845(2563-7284) | 124.127(64.412-186.041) | 25856(13827-38106) | 251.191(134.146-369.537) | 3.04 (2.83 to 3.25) |
| Ecuador | 9579(5489-13380) | 178.272(100.918-251.852) | 64179(35786-102752) | 395.547(219.911-629.738) | 2.61 (1.76 to 3.46) |
| Egypt | 73219(41529-118068) | 303.264(167.061-488.172) | 358342(202168-518336) | 611.765(335.914-896.363) | 2.52 (2.42 to 2.62) |
| El Salvador | 5896(3071-9210) | 192.135(99.469-301.884) | 38248(21021-58232) | 622.229(343.552-948.553) | 3.97 (3.5 to 4.44) |
| Equatorial Guinea | 355(170-597) | 172.236(84.081-287.286) | 2197(1013-3789) | 375.162(167.452-655.375) | 2.83 (2.47 to 3.19) |
| Eritrea | 383(156-746) | 31.352(12.67-59.248) | 1819(723-3650) | 62.771(25.898-124.178) | 2.35 (2.29 to 2.4) |
| Estonia | 1158(570-1807) | 58.562(28.729-91.695) | 3746(2025-5393) | 133.968(71.791-193.923) | 2.44 (2.04 to 2.84) |
| Eswatini | 572(294-903) | 203.701(102.381-327.387) | 2673(1292-4509) | 462.919(227.139-753.856) | 2.95 (2.26 to 3.64) |
| Ethiopia | 18977(8123-35408) | 94.126(41.27-174.485) | 38209(16973-67476) | 88.069(39.364-156.658) | -0.7 (-0.88 to -0.51) |
| Fiji | 735(358-1260) | 194.771(94.756-333.656) | 3199(1491-4956) | 427.121(200.313-667.406) | 2.19 (1.88 to 2.51) |
| Finland | 2282(1206-3476) | 32.421(17.038-49.547) | 5417(2706-8378) | 39.776(19.594-61.124) | 1.06 (0.9 to 1.22) |
| France | 34471(17383-52624) | 40.345(20.281-61.633) | 84765(43269-124178) | 50.946(26.236-74.319) | 0.86 (0.76 to 0.97) |
| Gabon | 1426(697-2254) | 248.493(119.972-389.257) | 5498(2517-9049) | 513.576(229.087-838.961) | 2.26 (2.03 to 2.49) |
| Gambia | 445(216-718) | 119.406(56.644-194.29) | 2272(1120-3851) | 211.041(104.206-358.343) | 1.62 (1.47 to 1.77) |
| Georgia | 3708(1944-5775) | 60.669(31.837-94.433) | 6471(3233-10427) | 115.296(57.62-185.771) | 2.4 (2.05 to 2.75) |
| Germany | 79521(39058-122566) | 62.604(30.697-96.215) | 180221(83148-289597) | 81.212(38.953-127.163) | 1.39 (1.18 to 1.6) |
| Ghana | 5377(2476-9151) | 83.913(38.03-140.658) | 41357(21244-67620) | 242.438(125.465-398.038) | 3.72 (3.63 to 3.81) |
| Greece | 16240(8644-23775) | 109.796(57.919-160.005) | 38071(20004-55684) | 136.706(72.692-199.198) | 0.43 (-0.19 to 1.05) |
| Greenland | 28(14-42) | 90.372(43.607-135.782) | 65(31-103) | 100.901(46.816-162.09) | 0.77 (0.61 to 0.92) |
| Grenada | 155(81-237) | 234.061(124.947-351.781) | 576(331-875) | 493.769(282.034-749.077) | 3.02 (2.82 to 3.22) |
| Guam | 131(69-197) | 175.321(90.511-265.136) | 511(261-790) | 243.922(126.48-372.963) | 1.75 (1.47 to 2.03) |
| Guatemala | 6686(3506-9959) | 193.076(100.583-283.816) | 46518(24254-72573) | 405.208(211.088-636.477) | 3.29 (2.84 to 3.74) |
| Guinea | 3265(1551-5565) | 97.209(45.81-166.263) | 8797(4057-15617) | 143.105(65.111-248.631) | 1.25 (1.2 to 1.31) |
| Guinea-Bissau | 607(278-1072) | 143.546(66.483-255.209) | 1631(721-2955) | 192.746(88.031-345.586) | 0.82 (0.77 to 0.88) |
| Guyana | 967(548-1419) | 231.123(129.367-348.171) | 3757(2071-5808) | 549.65(306.508-855.519) | 3.88 (3.52 to 4.23) |
| Haiti | 3127(1370-5837) | 86.364(38.115-158.661) | 12780(5227-26857) | 148.953(61.614-315.09) | 2.17 (2.03 to 2.3) |
| Honduras | 1926(978-3067) | 90.363(45.831-142.495) | 12364(6038-19654) | 190.671(92.39-308.336) | 2.68 (2.51 to 2.85) |
| Hungary | 8914(4979-13209) | 63.678(34.999-95.012) | 16017(8296-24494) | 82.515(42.922-126.583) | 1.43 (1.2 to 1.65) |
| Iceland | 102(57-149) | 34.624(19.17-50.247) | 304(165-442) | 49.578(27.237-72.264) | 1.32 (1.23 to 1.4) |
| India | 148738(72048-251785) | 29.44(14.217-49.362) | 838678(423437-1361373) | 67.595(33.91-108.539) | 2.81 (2.76 to 2.86) |
| Indonesia | 44127(19936-76039) | 40.122(17.635-69.001) | 237871(109018-390159) | 88.137(40.453-146.139) | 2.79 (2.64 to 2.93) |
| Iran (Islamic Republic of) | 23261(11908-37011) | 90.481(45.292-143.189) | 118152(62523-175045) | 154.933(80.866-230.657) | 1.81 (1.71 to 1.91) |
| Iraq | 26910(15221-40226) | 326.058(183.869-487.737) | 85118(41355-134695) | 362.314(179.214-589.427) | 0.01 (-0.12 to 0.15) |
| Ireland | 2536(1259-3845) | 63.835(31.415-96.663) | 6073(3310-8959) | 75.503(41.334-111.691) | 0.81 (0.69 to 0.92) |
| Israel | 5831(3032-8703) | 124.646(64.706-185.055) | 14938(7336-22942) | 114.383(56.319-175.569) | 0.12 (-0.24 to 0.49) |
| Italy | 48997(24766-73620) | 55.875(27.856-84.052) | 99727(49077-151090) | 58.165(28.76-87.457) | -0.01 (-0.11 to 0.09) |
| Jamaica | 2317(1138-3630) | 131.574(64.147-206.345) | 8680(4564-13133) | 277.743(146.629-420.797) | 2.03 (1.44 to 2.63) |
| Japan | 75752(37628-122971) | 46.082(22.798-74.839) | 181514(87201-291777) | 44.935(22.214-71.382) | -0.11 (-0.2 to -0.01) |
| Jordan | 3254(1828-4778) | 247.602(134.847-371.073) | 20905(12663-29795) | 300.636(178.462-429.764) | 0.39 (0.08 to 0.71) |
| Kazakhstan | 9742(4952-14834) | 74.415(38.186-113.015) | 21805(11167-33256) | 120.87(61.629-185.148) | 1.33 (1.08 to 1.58) |
| Kenya | 4778(2228-8365) | 56.765(27.084-100.863) | 34967(17243-55849) | 147.673(71.761-240.663) | 3.4 (3.31 to 3.5) |
| Kiribati | 80(37-131) | 201.94(91.31-337.922) | 270(118-489) | 354.121(154.057-645.523) | 1.7 (1.47 to 1.92) |
| Kuwait | 1489(839-2172) | 216.028(116.778-326.552) | 4824(2961-6756) | 161.287(95.134-228.321) | -0.76 (-0.98 to -0.54) |
| Kyrgyzstan | 2008(1102-3051) | 64.456(35.783-97.129) | 5857(3279-9050) | 111.886(61.663-169.651) | 1.07 (0.56 to 1.57) |
| Lao People's Democratic Republic | 1776(805-3225) | 81.479(36.392-149.409) | 5782(2306-11230) | 115.151(45.574-218.461) | 1.18 (1.12 to 1.24) |
| Latvia | 1407(758-2143) | 40.798(21.964-62.647) | 3115(1723-4663) | 80.585(43.87-122.457) | 2.2 (2.01 to 2.39) |
| Lebanon | 4221(2224-6925) | 199.395(102.965-325.648) | 15516(8553-23937) | 248.496(137.638-380.072) | 0.95 (0.76 to 1.14) |
| Lesotho | 682(325-1118) | 81.957(39.269-135.101) | 3186(1448-5446) | 291.25(130.859-498.388) | 5.1 (4.58 to 5.61) |
| Liberia | 2440(1258-4133) | 201.609(104.706-333.134) | 7864(3911-12831) | 306.696(151.254-514.655) | 1.63 (1.31 to 1.94) |
| Libya | 3160(1635-5145) | 162.853(83.293-267.558) | 22461(10893-35516) | 414.131(198.825-659.561) | 3.69 (3.47 to 3.9) |
| Lithuania | 1591(839-2474) | 36.196(18.884-56.319) | 3744(1998-5506) | 67.489(35.442-100.023) | 1.63 (1.44 to 1.82) |
| Luxembourg | 363(196-532) | 68.452(37.027-100.339) | 890(434-1397) | 78.755(38.911-121.769) | 0.73 (0.56 to 0.89) |
| Madagascar | 2017(883-3865) | 40.167(17.676-75.663) | 8233(3442-14739) | 73.729(30.806-131.909) | 1.98 (1.85 to 2.11) |
| Malawi | 3006(1367-5641) | 76.961(34.707-143.544) | 11452(5397-19873) | 149.544(71.456-256.177) | 1.95 (1.72 to 2.19) |
| Malaysia | 11732(5966-18815) | 119.615(61.382-189.722) | 59019(30263-91523) | 202.467(104.623-314.1) | 1.54 (1.38 to 1.7) |
| Maldives | 156(68-267) | 147.605(64.274-256.439) | 476(219-807) | 119.78(54.899-203.948) | -0.98 (-1.17 to -0.78) |
| Mali | 5118(2469-8629) | 123.667(60.295-210.605) | 14229(7387-23936) | 148.012(76.385-252.562) | 0.62 (0.46 to 0.77) |
| Malta | 266(130-407) | 65.038(31.955-99.204) | 808(379-1262) | 80.735(38.224-124.651) | 0.68 (0.44 to 0.93) |
| Marshall Islands | 36(15-69) | 206.253(85.386-410.026) | 150(39-376) | 391.801(99.743-1007.255) | 2.04 (1.82 to 2.26) |
| Mauritania | 2069(1018-3237) | 211.625(104.121-333.662) | 5771(2900-9593) | 275.898(135.919-446.621) | 0.51 (0.36 to 0.67) |
| Mauritius | 1466(713-2394) | 190.34(92.846-311.916) | 9634(4834-15444) | 518.928(257.867-830.501) | 3.52 (3.09 to 3.95) |
| Mexico | 103787(55042-157719) | 237.775(126.644-365.904) | 673923(392964-965604) | 517.361(301.278-743.751) | 3.02 (2.34 to 3.71) |
| Micronesia (Federated States of) | 120(58-211) | 239.414(114.591-431.245) | 367(161-607) | 456.856(191.807-770.657) | 2.12 (1.79 to 2.45) |
| Monaco | 40(23-60) | 55.067(31.72-81.785) | 84(45-123) | 79.98(44.184-116.611) | 1.32 (1.17 to 1.48) |
| Mongolia | 1344(724-2078) | 118.807(64.446-182.595) | 3407(1838-5342) | 131.638(71.641-203.133) | 0.05 (-0.07 to 0.18) |
| Montenegro | 715(409-1076) | 115.564(66.183-173.502) | 1513(819-2243) | 164.74(88.406-245.289) | 1.23 (1.11 to 1.35) |
| Morocco | 20462(10717-35503) | 141.494(73.855-249.51) | 93743(49431-149294) | 274.491(144.917-439.828) | 2.43 (2.32 to 2.55) |
| Mozambique | 2500(1011-4460) | 40.315(16.272-71.106) | 11787(5149-21472) | 95.333(40.846-177.073) | 3.24 (3.1 to 3.38) |
| Myanmar | 16191(6808-28752) | 63.981(27.271-112.568) | 41079(18889-72979) | 79.238(36.622-139.155) | 0.51 (0.43 to 0.6) |
| Namibia | 627(296-1070) | 96.354(45.1-164.138) | 2678(1359-4410) | 192.411(97.035-309.321) | 2.02 (1.68 to 2.36) |
| Nauru | 15(7-27) | 294.215(139.356-509.616) | 35(17-53) | 559.735(260.038-900.846) | 1.94 (1.81 to 2.07) |
| Nepal | 3742(1609-6561) | 34.705(15.069-59.482) | 19568(8985-33500) | 77.782(36.054-132.412) | 2.67 (2.39 to 2.95) |
| Netherlands | 8708(4118-13310) | 43.627(20.547-66.699) | 24823(12448-37094) | 67.332(33.801-100.155) | 1.47 (1.25 to 1.69) |
| New Zealand | 2259(1253-3291) | 59.423(32.867-86.755) | 7581(4387-10674) | 90.51(52.503-127.459) | 1.57 (1.23 to 1.91) |
| Nicaragua | 3322(1654-5174) | 200.727(98.923-312.369) | 25135(13188-40013) | 484.113(252.305-774.356) | 3.39 (3 to 3.78) |
| Niger | 2327(1110-3991) | 76.854(35.83-134.78) | 7556(3447-13124) | 84.498(39.084-147.771) | 0.17 (0.06 to 0.29) |
| Nigeria | 43300(20839-72318) | 100.218(48.046-165.186) | 163395(81568-266095) | 174.531(87.233-280.007) | 1.68 (1.59 to 1.77) |
| Niue | 5(2-8) | 212.643(99.456-351.628) | 10(4-18) | 469.779(195.428-844.954) | 2.54 (2.37 to 2.71) |
| North Macedonia | 1725(916-2641) | 92.737(49.373-143.173) | 3700(1870-5730) | 119.343(60.851-183.981) | 0.86 (0.65 to 1.07) |
| Northern Mariana Islands | 54(24-85) | 285.105(129.125-470.667) | 257(114-398) | 490.442(217.877-772.054) | 1.96 (1.78 to 2.14) |
| Norway | 2388(1227-3655) | 35.079(18.042-53.766) | 4988(2600-7490) | 46.973(24.424-70.461) | 1.07 (0.93 to 1.21) |
| Oman | 874(409-1484) | 120.416(54.912-199.361) | 7497(4494-11199) | 362.051(209.963-540.452) | 4.33 (4.07 to 4.58) |
| Pakistan | 41147(20124-68430) | 69.848(34.131-117.151) | 216552(104173-362496) | 154.489(72.489-260.34) | 2.56 (2.34 to 2.78) |
| Palau | 22(11-37) | 227.997(113.924-378.916) | 95(45-151) | 427.14(204.4-687.949) | 2.18 (1.99 to 2.36) |
| Palestine | 2281(1239-3652) | 272.943(145.841-445.295) | 7907(4479-11831) | 328.543(176.648-496.524) | 0.53 (0.34 to 0.71) |
| Panama | 1731(913-2553) | 116.981(62.089-171.112) | 12452(6756-18346) | 280.266(152.026-412.746) | 3.1 (2.74 to 3.47) |
| Papua New Guinea | 957(382-1834) | 46.413(18.769-89.764) | 3502(1364-6586) | 57.672(23.431-110.841) | 0.6 (0.54 to 0.66) |
| Paraguay | 3287(1857-4775) | 146.73(81.827-212.699) | 14747(8040-21735) | 249.967(135.172-369.659) | 1.85 (1.74 to 1.97) |
| Peru | 21000(10850-30280) | 175.205(89.539-255.234) | 86721(48392-128306) | 256.887(142.45-384.346) | 1.02 (0.83 to 1.22) |
| Philippines | 21407(9663-36895) | 67.278(30.168-116.933) | 141259(60309-246424) | 158.856(66.989-274.381) | 3.21 (3.03 to 3.39) |
| Poland | 43585(23725-64620) | 101.485(54.823-151.423) | 45940(25901-70098) | 65.096(36.89-99.402) | -1.5 (-1.89 to -1.11) |
| Portugal | 10202(5445-15546) | 77.425(41.161-119.323) | 24005(12346-37109) | 87.16(46.05-132.635) | 0.46 (0.05 to 0.88) |
| Puerto Rico | 9737(5823-13929) | 275.109(164.339-392.768) | 22862(13591-33424) | 343.356(209.296-487.615) | 1.36 (1.01 to 1.71) |
| Qatar | 286(143-484) | 251.275(122.496-447.016) | 3116(1928-4450) | 357.487(216.838-516.584) | 0.84 (0.29 to 1.4) |
| Republic of Korea | 13318(6126-22851) | 49.487(22.147-85.17) | 38495(18372-64832) | 41.456(19.822-69.7) | -0.31 (-0.45 to -0.17) |
| Republic of Moldova | 1884(1045-2922) | 44.455(24.262-68.709) | 4520(2655-6726) | 77.427(45.771-115.612) | 1.6 (1.45 to 1.76) |
| Romania | 24564(13693-36766) | 91.933(51.229-138.447) | 36202(21623-52574) | 102(60.532-148.113) | 0.74 (0.44 to 1.05) |
| Russian Federation | 79384(41550-120343) | 45.418(23.663-69.13) | 140722(77923-204665) | 59.787(33.327-87.436) | 0.41 (0.21 to 0.62) |
| Rwanda | 2079(829-3919) | 70.151(29.373-134.073) | 5312(2178-9946) | 83.3(33.834-156.243) | -0.38 (-0.77 to 0.02) |
| Saint Kitts and Nevis | 110(60-170) | 319.777(178.17-475.692) | 366(205-531) | 517.713(289.913-751.22) | 2.35 (2.05 to 2.66) |
| Saint Lucia | 193(100-290) | 222.706(116.671-336.21) | 815(441-1232) | 342.302(185.13-513.726) | 1.86 (1.6 to 2.11) |
| Saint Vincent and the Grenadines | 99(48-159) | 139.973(68.677-223.555) | 438(234-671) | 314.739(169.647-483.75) | 3.28 (2.99 to 3.58) |
| Samoa | 212(101-349) | 239.444(112.616-399.717) | 599(289-912) | 406.794(193.533-625.242) | 1.71 (1.64 to 1.78) |
| San Marino | 17(9-25) | 47.058(25.951-69.104) | 39(21-61) | 47.456(25.363-71.977) | 0.59 (0.4 to 0.77) |
| Sao Tome and Principe | 130(65-205) | 204.94(101.501-323.5) | 495(249-779) | 425.18(210.623-659.72) | 2.27 (2.16 to 2.38) |
| Saudi Arabia | 19530(10277-31223) | 307.605(158.41-501.405) | 170261(100966-245817) | 764.256(444.937-1126.37) | 2.69 (2.47 to 2.91) |
| Senegal | 5125(2562-8213) | 153.201(76.21-245.87) | 16014(7889-27119) | 198.514(95.511-333.455) | 0.65 (0.53 to 0.77) |
| Serbia | 10962(5826-17197) | 105.045(56.596-164.996) | 20786(11038-32090) | 126.438(67.24-193.749) | 0.5 (0.41 to 0.6) |
| Seychelles | 99(51-154) | 175.732(91.525-273.354) | 422(212-666) | 352.833(173.918-553.447) | 2.41 (2.2 to 2.63) |
| Sierra Leone | 1788(841-3105) | 87.724(41.985-151.771) | 4926(2400-8317) | 121.196(59.717-204.73) | 1.01 (0.98 to 1.04) |
| Singapore | 971(472-1604) | 44.401(21.457-73.747) | 4244(2029-6647) | 49.817(23.812-78.765) | 1.55 (1.13 to 1.97) |
| Slovakia | 6102(3538-9041) | 103.114(59.36-152.17) | 8702(4968-12399) | 93.268(53.507-133.813) | -0.33 (-0.37 to -0.29) |
| Slovenia | 1508(832-2254) | 62.179(34.288-93.602) | 2947(1636-4319) | 64.155(35.822-94.154) | 0.27 (0.12 to 0.42) |
| Solomon Islands | 172(61-364) | 110.232(41.532-232.719) | 507(185-972) | 125.017(45.264-244.131) | 0.15 (-0.04 to 0.34) |
| Somalia | 2052(899-4065) | 76.303(34.676-149.788) | 8345(3675-15756) | 120.854(53.013-226.238) | 1.57 (1.5 to 1.65) |
| South Africa | 28089(14580-43834) | 125.345(64.93-196.604) | 130631(70383-194853) | 276.153(147.09-415.815) | 2.48 (2.12 to 2.84) |
| South Sudan | 1347(598-2803) | 52.603(23.252-109.416) | 4146(1716-7809) | 102.999(42.699-199.819) | 2.11 (1.85 to 2.36) |
| Spain | 41260(20801-62967) | 77.309(38.914-118.157) | 78900(38166-122752) | 68.563(34.563-104.008) | -0.18 (-0.28 to -0.08) |
| Sri Lanka | 6817(2948-12160) | 60.775(26.54-107.281) | 24231(10145-45405) | 90.413(37.62-170.675) | 1.22 (1.01 to 1.42) |
| Sudan | 11972(5963-21048) | 124.446(61.63-219.497) | 51229(24864-83497) | 244.499(116.844-407.583) | 2.24 (2.06 to 2.42) |
| Suriname | 500(263-765) | 181.523(95.093-282.667) | 2205(1130-3553) | 340.88(174.786-548.992) | 2.29 (2.1 to 2.47) |
| Sweden | 4814(2434-7317) | 31.258(15.822-47.723) | 12465(6275-19485) | 50.756(25.833-78.558) | 1.98 (1.85 to 2.1) |
| Switzerland | 4901(2361-7614) | 45.96(22.172-71.112) | 10703(4782-17843) | 50.776(23.118-83.485) | 0.82 (0.62 to 1.01) |
| Syrian Arab Republic | 13389(7286-21213) | 252.825(137.033-402.481) | 51095(28678-78861) | 409.14(221.456-633.811) | 1.18 (0.9 to 1.45) |
| Taiwan | 15137(6775-26061) | 105.636(47.113-183.585) | 51722(23461-86673) | 119.574(54.572-200.623) | 0.97 (0.72 to 1.22) |
| Tajikistan | 1329(723-2070) | 46.875(25.374-72.776) | 3842(2077-5953) | 60.837(33.011-94.066) | 0.7 (0.62 to 0.79) |
| Thailand | 22564(9623-40197) | 60.903(26.359-109.192) | 154844(67202-273661) | 143.125(61.957-251.583) | 2.47 (2.26 to 2.69) |
| Timor-Leste | 92(36-179) | 29.744(11.843-57.517) | 435(182-863) | 49.666(20.933-100.335) | 1.95 (1.62 to 2.29) |
| Togo | 1259(616-2078) | 100.039(48.586-167.284) | 6471(3169-11121) | 169.146(82.372-281.247) | 1.55 (1.48 to 1.62) |
| Tokelau | 2(1-4) | 156.921(73.037-291.529) | 4(2-7) | 288.169(129.303-474.985) | 2 (1.86 to 2.15) |
| Tonga | 76(35-122) | 129.889(58.984-213.285) | 181(82-276) | 224.72(100.252-343.513) | 1.62 (1.46 to 1.79) |
| Trinidad and Tobago | 1533(824-2236) | 178.466(95.203-263.351) | 7867(4236-12355) | 409.816(221.064-640.435) | 3.22 (2.9 to 3.55) |
| Tunisia | 4819(2407-7913) | 96.191(47.286-160.256) | 26998(13421-43497) | 208.961(104.082-338.118) | 2.57 (2.54 to 2.6) |
| Turkey | 68105(37505-105726) | 203.359(109.719-317.439) | 217896(122907-323605) | 239.133(134.14-356.892) | 0.88 (0.57 to 1.19) |
| Turkmenistan | 1983(1049-3108) | 94.269(50.087-144.08) | 8118(4348-13273) | 182.404(96.882-297.577) | 2.05 (1.72 to 2.39) |
| Tuvalu | 11(4-20) | 146.718(57.882-282.298) | 30(11-55) | 282.429(108.106-518.397) | 2.18 (2.09 to 2.26) |
| Uganda | 3631(1535-6695) | 54.813(23.326-101.408) | 16122(7248-29425) | 102.612(45.942-188.32) | 1.63 (1.43 to 1.83) |
| Ukraine | 15799(8610-24421) | 23.62(12.821-36.403) | 33513(18340-51044) | 46.235(25.112-71.136) | 2.64 (2.38 to 2.91) |
| United Arab Emirates | 957(542-1585) | 159.132(81.782-268.106) | 12556(7186-17621) | 356.876(184.272-506.282) | 4.48 (3.89 to 5.07) |
| United Kingdom | 37231(17924-56602) | 42.516(20.478-65.049) | 67528(32798-105294) | 51.341(25.557-79.663) | 0.86 (0.75 to 0.97) |
| United Republic of Tanzania | 10136(5049-16938) | 92.272(44.264-154.522) | 45260(23394-76212) | 170.052(88.621-285.335) | 2 (1.95 to 2.04) |
| United States of America | 265590(145681-369143) | 84.533(46.765-116.932) | 1286175(742874-1769939) | 226.761(134.118-307.136) | 0.97 (0.75 to 1.18) |
| United States Virgin Islands | 182(108-254) | 204.018(118.218-291.511) | 378(210-568) | 245.182(142.129-357.244) | 3.52 (3.32 to 3.73) |
| Uruguay | 3111(1525-4593) | 80.041(39.08-117.419) | 6222(3060-9450) | 108.272(54.653-161.373) | 1.24 (1.02 to 1.46) |
| Uzbekistan | 7741(4165-11673) | 66.027(35.373-100.841) | 42191(21353-65353) | 146.398(74.222-228.215) | 2.17 (1.7 to 2.64) |
| Vanuatu | 88(41-167) | 135.954(63.191-256.333) | 412(183-748) | 218.269(96.255-401.516) | 1.47 (1.41 to 1.52) |
| Venezuela (Bolivarian Republic of) | 14940(8097-22198) | 146.461(79.1-216.457) | 108473(59122-166724) | 356.895(192.531-548.259) | 2.51 (2.11 to 2.91) |
| Viet Nam | 9705(4159-18713) | 24.221(10.314-46.637) | 50039(20022-93454) | 49.539(19.567-91.316) | 2.84 (2.6 to 3.08) |
| Yemen | 3766(1827-6989) | 75.125(36.69-143.472) | 17781(8734-30816) | 124.164(60.27-219.309) | 1.54 (1.35 to 1.73) |
| Zambia | 2883(1330-5113) | 98.063(44.823-173.572) | 16186(7627-29905) | 210.318(100.539-373.557) | 2.12 (1.95 to 2.3) |
| Zimbabwe | 3099(1384-5306) | 74.786(33.833-128.286) | 16619(7732-28424) | 224.676(103.567-381.462) | 4.03 (3.45 to 4.62) |

DALYs: disability-adjusted life years.
